# DNA origami–based artificial antigen-presenting cells for adoptive T cell therapy

**DOI:** 10.1126/sciadv.add1106

**Published:** 2022-12-02

**Authors:** Yueyang Sun, Jiajia Sun, Mingshu Xiao, Wei Lai, Li Li, Chunhai Fan, Hao Pei

**Affiliations:** ^1^ Shanghai Key Laboratory of Green Chemistry and Chemical Processes, School of Chemistry and Molecular Engineering, Shanghai Frontiers Science Center of Genome Editing and Cell Therapy, East China Normal University, 500 Dongchuan Road, Shanghai 200241, China.; ^2^ School of Chemistry and Chemical Engineering, and Institute of Molecular Medicine, Renji Hospital, School of Medicine, Shanghai Jiao Tong University, Shanghai 200240, China.

## Abstract

Nanosized artificial antigen-presenting cells (aAPCs) with efficient signal presentation hold great promise for in vivo adoptive cell therapy. Here, we used DNA origami nanostructures as two-dimensional scaffolds to regulate the spatial presentation of activating ligands at nanoscale to construct high-effective aAPCs. The DNA origami–based aAPC comprises costimulatory ligands anti-CD28 antibody anchored at three vertices and T cell receptor (TCR) ligands peptide–major histocompatibility complex (pMHC) anchored at three edges with varying density. The DNA origami scaffold enables quantitative analysis of ligand-receptor interactions in T cell activation at the single-particle, single-molecule resolution. The pMHC-TCR–binding dwell time is increased from 9.9 to 12.1 s with increasing pMHC density, driving functional T cell responses. In addition, both in vitro and in vivo assays demonstrate that the optimized DNA origami–based aAPCs show effective tumor growth inhibiting capability in adoptive immunotherapy. These results provide important insights into the rational design of molecular vaccines for cancer immunotherapy.

## Teaser

A DNA origami–based artificial antigen-presenting cell is constructed for adoptive T cell therapy.

## INTRODUCTION

Adoptive T cell immunotherapy is a promising medical strategy for cancer treatment by transfusion of ex vivo functional T cells (*1*–*3*). Essential to this approach is the efficient activation and massive expansion of T cells to enhance the patient’s immune response (*4*–*6*). Various artificial antigen-presenting cells (aAPCs) have been developed as promising alternatives to natural APCs for T cell activation and expansion (*7*, *8*). In general, aAPCs are constructed by integrating T cell receptor (TCR) ligands [e.g., peptide–major histocompatibility complex (pMHC) and anti-CD3 antibody] and costimulatory ligands (e.g., anti-CD28 and 4-1BBL antibodies) on the surface of biocompatible materials, including liposomes, exosomes, polymers, magnetic microbeads, and mesoporous silica micro-rods (*9*–*13*). The nanoscale organization of ligands is known to be critical for triggering intracellular signaling involved in T cell activation (*14*–*16*), however inherently challenging to investigate. Arrays of gold nanoparticle–bound TCR ligands have been used to study the impact of the spatial organization of ligands (e.g., ligand orientation, surface density, and lateral spacing) on TCR triggering (*17*). Those arrays, fabricated by electron beam lithography or block copolymer micellar nanolithography, enable the indirect regulation of ligand spacing through varying lateral spacing of gold nanoparticles, which only allowed statistical control over the positions and numbers of ligands (*5*, *17*–*19*). In addition, despite that these two-dimensional (2D) nanofabricated surfaces are proven useful in elucidating the mechanisms of T cell activation, currently, they are less suitable as “off-the-shelf” aAPCs since they cannot eliminate the need for time-consuming and expensive isolation and culture of autologous APCs.

DNA origami nanostructures could provide an ideal scaffold to organize ligands with nanoscale addressability for engineering high-effective aAPCs for adoptive T cell therapy (*20*–*24*). The inherent Watson-Crick pairing specificity of DNA hybridization allows precise assembly of different biomolecules (e.g., nucleic acids, proteins, ligands, and receptors) (*25*–*27*) following a prescribed path without interfering with each other, thus ensuring control over ligand valency at the single-molecule level (*28*, *29*). In addition, the site-specific addressability of DNA origami nanostructures allows precise anchoring of discrete ligands on prescribed positions, thus ensuring control over ligand distance with nanometer precision. Furthermore, DNA origami nanostructures by their very nature have excellent in vivo properties. In two recent studies, DNA origami nanostructures have been used to interrogate the impact of nanoscale antigen organization on the early activation of calcium signaling involved in T cell or B cell activation (*30*, *31*). Exploiting DNA origami nanostructures to spatially organize ligands on nanoscale thus holds great potential for manipulating immune stimulation in vivo. One recent study by Ding and co-workers (*29*) used DNA origami nanostructures to load the tumor antigen peptide/CpG loop/double-stranded RNA to construct a DNA nanodevice–based vaccine for cancer immunotherapy in vivo.

Here, we introduced a triangular DNA origami as a 2D scaffold to regulate the spatial organization of activating ligands on nanoscale to enable optimized T cell activation for adoptive immunotherapy. The costimulatory ligands anti-CD28 antibody (aCD28) were anchored at three vertices with fixed valency of three copies, while TCR ligands pMHC were anchored at three edges on DNA origami with varying pMHC density. We showed that pMHC organization on DNA origami with high density would be favorable for more effective CD8^+^ T cell activation, defined by CD69 up-regulation, interferon-γ (IFN-γ) secretion, and cell proliferation. This allows quantitative analysis of ligand-receptor interactions in T cell activation at the single-particle, single-molecule resolution using total internal reflection fluorescence (TIRF) microscopy. Our findings revealed that pMHC-TCR binding dwell time was increased from 9.9 to 12.1 s with increasing pMHC density, thus substantially driving functional T cell responses. Furthermore, both in vitro and in vivo assays demonstrated that the optimized DNA origami–based aAPCs can effectively stimulate T cell proliferation and inhibit tumor growth with adoptive immunotherapy.

## RESULTS

### Design of DNA origami–based aAPCs

**Fig. 1.**
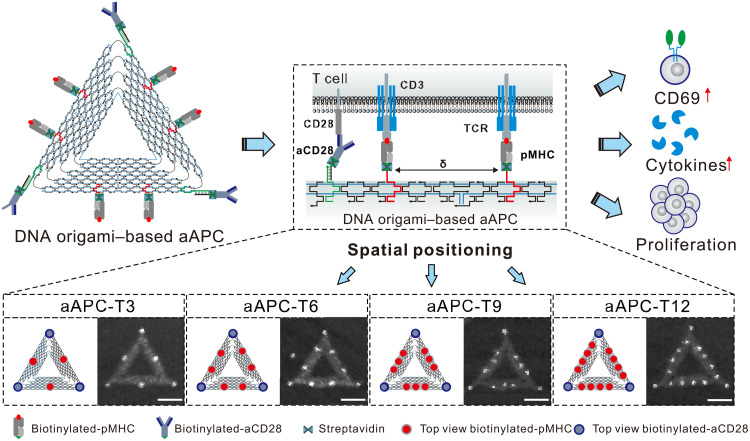
Design of DNA origami–based aAPCs. DNA origami functionalized with ligands (pMHC and aCD28) at predefined positions set ligand copy number and spacing (δ). Here, four types of DNA origami–based aAPCs were designed for tuning T cell activation (denoted as aAPC-T*n*; *n* indicates the number of SA-binding site), including aAPC-T3, aAPC-T6, aAPC-T9, and aAPC-T12. Scale bars, 50 nm.

We fabricated triangular DNA origami nanostructures (edge length, ~120 nm) with multiple functional elements as the 2D scaffolds to spatially control the assembly of pMHC ligands and aCD28 (Fig. 1). For pMHC organization, biotinylated DNA origami was first fabricated by labeling-specific stable strands with biotins. The biotins were modified at predesigned sites on assembled DNA origami, serving as streptavidin (SA)–binding site to enable site-specific anchoring of biotinylated pMHC monomer via biotin-SA conjugation (*32*), yielding pMHC-modified DNA origami (denoted as pMHC-T*n*; *n* indicates the number of SA-binding site). In this design, each SA-binding site can anchor three pMHC molecules. Next, DNA-ligated aCD28 was site-specifically coupled to pMHC-modified DNA origami via hybridization to overhang strands extended at the vertex (*29*, *33*). This geometrical arrangement is based on previous studies, demonstrating that presenting costimulatory ligands around TCR ligands could be more effective in enhancing T cell function (*34*). Using this approach, we designed four types of DNA origami–based aAPCs (denoted as aAPC-T*n*; *n* indicates the number of SA-binding site, *n* = 3, 6, 9, and 12) that present the aCD28 in copy numbers of 3 at three vertices and the pMHC molecules in copy numbers varying from 9 to 36 with inter-pMHC spacing varying from 60 to 20 nm (Fig. 1, bottom).

To validate our design, we characterized the stepwise assembly of DNA origami–based aAPCs by atomic force microscopy (AFM) imaging and agarose gel (AGE) analysis (figs. S1 to S5). AFM imaging demonstrated that pMHC molecules and aCD28 were both successfully bound to the DNA origami at specific programmed locations, which was also confirmed by the increased cross-sectional height after each assembly step (fig. S6). In addition, AGE analysis confirmed that DNA origami–based aAPCs preserved their structural integrity for at least 72 hours in phosphate-buffered saline (PBS) and 10% fetal bovine serum (FBS) (figs. S7 and S8). Meanwhile, DNA origami–based aAPCs induced little negative influence on cell viability at concentrations ranging from 5 to 20 nM, indicating their excellent biocompatibility (fig. S9).

### Effect of ligand organization on in vitro stimulation of CD8^+^ T cell effectors

**Fig. 2.**
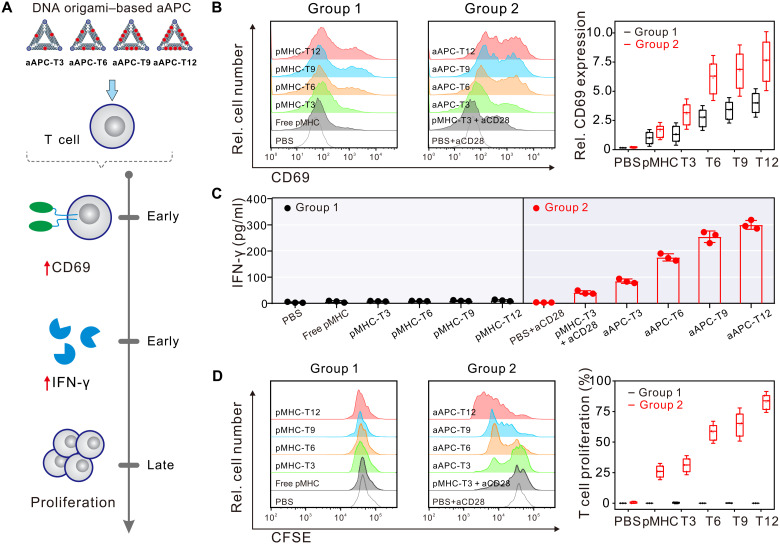
T cell activation by DNA origami–based aAPCs. (**A**) Work flow diagram depicting that T cell activation by DNA origami–based aAPCs was assayed by analyzing CD69 up-regulation, cytokine secretion, and cell proliferation. (**B**) Flow cytometry analysis of CD69 expression of T cells after stimulation by DNA origami–based aAPCs and their corresponding relative CD69 expression (ratio of percentage of CD69^+^ cells of each group to that of free pMHC). Data are shown as means ± SD (*n* = 3). (**C**) Quantitative analysis of IFN-γ levels secreted by T cells after stimulation by DNA origami–based aAPCs. Data are shown as means ± SD (*n* = 3). (**D**) Flow cytometry analysis of CD8^+^ T cell proliferation after stimulation by DNA origami–based aAPCs and their corresponding CD8^+^ T cell proliferation. Group 1, pMHC-T*n*; group 2, aAPC-T*n*; control groups, free pMHC, free aCD28, and pMHC-T3 + free aCD28. Data are shown as means ± SD (*n* = 3).

We next investigated the stimulatory potency of DNA origami–based aAPCs by incubation with CD8^+^ T cells isolated from OT-1 mice (Fig. 2A). Previous work unraveled that Ca^2+^ release from the endoplasmic reticulum is one of the earliest activation events in pMHC-TCR triggering and the subsequent calcium influx through Ca^2+^ release–activated Ca^2+^ channels leads to an increase in cytosolic Ca^2+^ concentration (*35*). Here, we used Ca^2+^-sensitive fluorescent dye (i.e., Fura-4) for the detection of intracellular calcium signaling of CD8^+^ T cells after 1 hour of incubation with DNA origami–based aAPCs presenting ovalbumin 257-264 (OVA_257–264_)–specific antigens. Control experiments showed that stimulated T cells exhibited a substantial increase in calcium signaling (fig. S10), in which calcium signaling activity was found to monotonically increase with increasing pMHC density and plateaued with aAPC-T12 (fig. S11). We then monitored several representative short-term and long-term indicators of cellular activation, including CD69 up-regulation, cytokine secretion, and cell proliferation. After short-term (16 hours) incubation with CD8^+^ T cells, pMHC-T3 without costimulatory ligands (i.e., aCD28) could activate CD69 expression (Fig. 2B, left, group 1), and their corresponding percentage of CD69^+^ cells was found to positively correlate with the pMHC density on DNA origami, in which CD69 expression on T cells stimulated by pMHC-T12 is more than fourfold of that stimulated by free pMHC (Fig. 2B, right). As expected, DNA origami–based aAPCs that simultaneously provide TCR stimulation and costimulatory signals enable further marked up-regulation of CD69 expression (Fig. 2B, middle, group 2). More specifically, CD69 expression on T cells stimulated by aAPC-T3, aAPC-T6, aAPC-T9, and aAPC-T12 was 3.0-fold, 6.1-fold, 6.8-fold, and 7.6-fold of that stimulated by free pMHC (Fig. 2B, right). We next investigated the secretion of IFN-γ from CD8^+^ T cells. We found that stimulating T cells with pMHC-T*n* or free pMHC cannot induce obvious IFN-γ production (Fig. 2C, left, group 1). In contrast, IFN-γ secretion could only be triggered upon engagement of both pMHC and aCD28 in stimulating T cells (Fig. 2C, right, group 2). These results disclose that complete T cell activation requires both TCR triggering and costimulation signal, which agrees well with the generally accepted two-signal model of lymphocyte activation (*36*, *37*). The IFN-γ production also increased monotonically with increasing pMHC density and plateaued with aAPC-T12 (up to ~300 ng/ml) (Fig. 2C, right, group 2), similar to the trend observed in CD69 up-regulation (Fig. 2B). aAPC-T3 could induce twofold CD69 expression and twofold IFN-γ production compared with pMHC-T3 + aCD28 (that is the mixture of pMHC-T3 and free aCD28), implying the importance of multiligand arrangement to T cell activation. Moreover, we compared the CD69 expression after stimulation by DNA origami–based APCs with the same pMHC number and different inter-pMHC spacing, including 20-nm aAPC-T6, 40-nm aAPC-T6, 60-nm aAPC-T6, and 80-nm aAPC-T6 (fig. S12). The results showed that the shorter inter-pMHC spacing on the DNA origami tends to induce stronger T cell activation in terms of the percentage of CD69^+^ cells. In addition, to investigate the potential impact of geometry of DNA origami scaffold on T cell triggering, we designed a cross DNA origami–based aAPC (fig. S13A) to compare cellular activation with the triangular DNA origami–based aAPC. In the cross DNA origami–based aAPC, pMHC and aCD28 were assembled on the same side of a cross origami platform, in which the copy numbers of pMHC and aCD28 were the same as those of aAPC-T12 and the inter-pMHC spacing at each edge of cross origami was 20 nm (fig. S13A). The results showed that the cross DNA origami–based APC led to weaker T cell activation in terms of the percentage of CD69^+^ cells, which is likely due to more crowded arrangement of ligands on cross DNA origami leading to larger steric hindrance (fig. S13B).

Next, cell proliferation was evaluated after a long-term (3 days) of culturing using carboxyfluorescein diacetate succinimidyl ester (CFSE; a dye for labeling intracellular proteins) (*38*). We observed increasing percentage of the original T cells entering proliferation with increasing pMHC density on aAPCs (up to 83% for aAPC-T12) and a slightly higher percentage in aAPC-T3 than that in pMHC-T3 + aCD28 (~26%), in line with the observations in CD69 up-regulation and IFN-γ production. Meanwhile, we observed clusters of activated T cells, confirming that DNA origami–based aAPCs well support T cell survival on a longer time scale (fig. S14). We also found that pMHC-T*n* alone or free pMHC exhibited weak capability of inducing CD8^+^ T cells proliferation (Fig. 2D, left, group 1), whereas aAPC-T*n* and pMHC-T3 + aCD28 could efficiently trigger proliferation of CD8^+^ T cells (Fig. 2D, middle, group 2), further confirming that the coexistence of both pMHC and aCD28 is required for effective activation of CD8^+^ T cells. Overall, these results demonstrate that T cell activation, defined by CD69 up-regulation, IFN-γ production, and cell proliferation, correlated closely with both pMHC density and multiligand organization, in which aAPC-T12 enabled the most effective T cell activation.

### Mapping individual DNA origami–based aAPC-TCR binding events to cellular activation

**Fig. 3.**
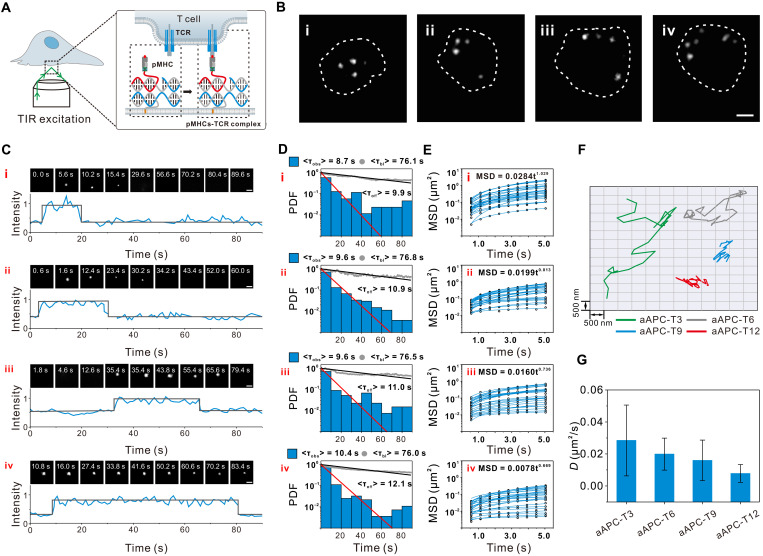
Real-time monitoring of single DNA origami–based aAPC-TCR–binding events. (**A**) Scheme of TIRF imaging of the intermembrane junction between plasma membrane and SLBs. Within the lipid bilayer, freely mobile DNA origami–based aAPCs experience rapid diffusion and were blurred out under imaging, whereas TCR-ligated DNA origami–based aAPCs experience reduced mobility and were shown as discrete puncta. (**B**) Localization of bound single DNA origami–based aAPC using long exposure times and low-power intensity imaging under a T cell (dashed line). Scale bar, 3 μm. i, aAPC-T3; ii, aAPC-T6; iii, aAPC-T9; iv, aAPC-T12. (**C**) Single DNA origami–based aAPC-TCR–binding and unbinding events over time determined by TIRF. Images (top) and intensity traces (bottom) are representative of 15 independent experiments. i, aAPC-T3; ii, aAPC-T6; iii, aAPC-T9; and iv, aAPC-T12. (**D**) Dwell time of DNA origami–based aAPCs using long exposure and low-power intensity imaging at a 560-ms interval and corrected for fluorophore photobleaching (gray). The distribution of observed dwell times (<τ_obs_>) was determined after data fitting (red lines). Bleaching times (<τ_bl_>) (gray circle) were measured using DNA origami–based aAPCs with the same fluorescent label (AF488-labeled DNA) and without cells. Bleaching times (<τ_bl_>) were determined after data fitting (black lines). Probability density function (PDF) plot were obtained from the analysis of >50 trajectories from >10 cells and are representatives of three independent experiments. (**E**) Time-dependent MSD plots of DNA origami–based aAPCs on SLBs obtained from 25 trajectories in three independent experiments. The diffusion coefficient (*D*) was determined after data fitting (blue lines) from each particle trajectory. (**F**) Moving trajectories of DNA origami–based aAPCs. (**G**) Average diffusion coefficient (*D*) of DNA origami–based aAPCs, as determined by single-particle tracking and MSD analysis from 25 trajectories in (F). Data are shown as means ± SD (*n* = 3).

To gain deep insight into efficient T cell activation by DNA origami–based aAPCs, we used TIRF microscopy for tracking the DNA origami–based aAPC-TCR–binding events experienced by T cells at the single-particle and single-cell level (*39*). In this experiment, supported lipid bilayers (SLBs) were first functionalized with histidine-tagged intercellular adhesion molecule–1 (ICAM-1) and DNA origami–based aAPCs [labeled with three Alexa Fluor 488 (AF488)–labeled DNA] through insertion of cholesterol into phospholipids membrane (*40*). Fluorescence recovery after photobleaching (FRAP) characterization demonstrated that the constructed SLBs had good fluidity and integrality (fig. S15). After exposing T cells to the functionalized SLBs, an essentially planar junction was formed between individual T cells and SLBs due to the interaction between leukocyte function–associated antigen 1 and ICAM-1 (Fig. 3A). This interaction initiated T cell spreading and was followed by pMHC binding to TCRs at the interface. To discriminate the bound aAPC-TCR from free aAPCs, we conducted TIRF imaging under long exposure times (280 ms) and low laser illumination intensity (0.1 mW/cm^2^). Under this imaging condition, only the TCR-bound aAPCs could be detected as discrete puncta, whereas fast-moving, free aAPCs would be blurred out (*41*). As displayed in Fig. 3B, more TCR-bound DNA origami–based aAPCs could be clearly observed with increasing pMHC density owing to the enhanced receptor-ligand–binding efficiency.

Next, the dwell time of aAPC-T*n* binding to T cells was analyzed. Using the low-power, long-exposure strategy, the binding dwell time could be resolved at the single-particle level by single-step intensity loss observations (Fig. 3C). As shown in Fig. 3 (C and D), we observed long-binding kinetics with mean dwell times in tens of seconds (Fig. 3, C and D), which could be attributed to the multivalent binding of DNA origami–based aAPCs to T cells. Moreover, the mean dwell times (<τ_off_>) increased from 9.9 to 12.1 s with increasing pMHC density (Fig. 3D), and these long dwell events would facilitate T cell activation (*41*).

To further investigate the diffusion of aAPCs on T cell surface, we monitored the moving trajectory of DNA origami–based aAPCs. With increasing the pMHC density, the 2D diffusion of DNA origami–based aAPCs became more stationary (Fig. 3, E and F). Their diffusion coefficient (*D*) can be calculated on the basis of Eq. 1 (*42*, *43*)

$$\text{MSD}=4D\;t^{\alpha }$$ (1)

where MSD is the mean square displacement of lateral motion of an aAPC, *t* is the observed lag time, and α is the exponent factor carrying information about the motion type. The resulting MSD plots displayed an almost linear increase over time from 25 tracked aAPCs (Fig. 3E). From the MSD analysis, the average α drops from 1.029 ± 0.269 (corresponding to aAPC-T3 carrying 9 copies of pMHC) to 0.699 ± 0.328 (corresponding to aAPC-T12 carrying 36 copies of pMHC) (fig. S16). The obtained α indicates the diffusion behavior of aAPCs changing from Brownian motion to subdiffusion on the T cell surface, which agrees well with previous work (*44*). Moreover, the diffusion coefficient (*D*) of DNA origami–based aAPCs also decreases from 0.028 ± 0.022 μm^2^/s (aAPC-T3) to 0.008 ± 0.005 μm^2^/s (aAPC-T12) (Fig. 3G). Moreover, the number of large aAPCs-TCR complex clusters increased with increasing the pMHC density, which is consistent with a previous report that TCRs in dense clusters have the highest signaling efficiency (fig. S17) (*16*). All these observations suggest that increasing pMHC density indeed increases the aAPC-TCR–binding dwell time and slows the diffusivity of aAPCs, leading to efficient T cell activation.

### In vitro cancer cell–specific killing of DNA origami–based aAPCs–activated T cells

**Fig. 4.**
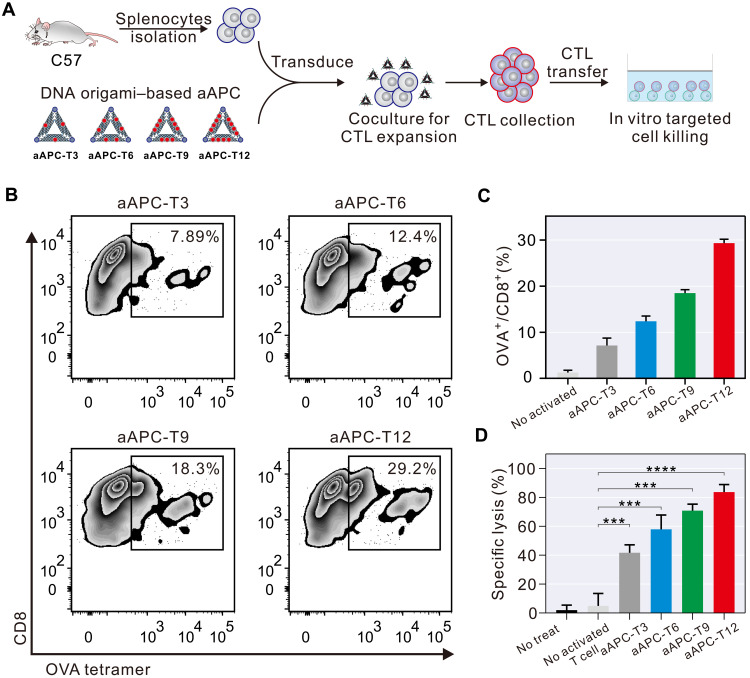
Splenocytes stimulated with DNA origami–based aAPCs enhance in vitro targeted cancer cell killing. (**A**) Work flow diagram depicting the T cell activation with DNA origami–based aAPCs for in vitro targeted cancer cell killing. (**B**) Flow cytometry analysis of OVA_257–264_–specific CD8^+^ T cell populations in the splenocytes stimulated by DNA origami–based aAPCs, including aAPC-T3, aAPC-T6, aAPC-T9, and aAPC-T12. (**C**) Plots summarizing the percentage of CD8^+^ T cells in the splenocytes stimulated by DNA origami–based aAPCs, including aAPC-T3, aAPC-T6, aAPC-T9, and aAPC-T12. Data are shown as means ± SD (*n* = 3). (**D**) Cytotoxic activity of splenocytes stimulated by DNA origami–based aAPCs toward B16-OVA cells. Data are shown as means ± SD (*n* = 3). Two-tailed *t* test was used to calculate *P* values. ***P < 0.001 and ****P < 0.0001.

To evaluate the therapeutic potential, we examined in vitro targeted cancer cell–killing efficacy of the stimulated T cells by DNA origami–based aAPCs. The splenocytes from C57 mice were cocultured with DNA origami–based aAPCs presenting OVA_257–264_–specific antigens for 3 days. The stimulated splenocytes were then incubated with melanoma cells expressing the OVA antigen (B16-OVA) (Fig. 4A). Through the tetramer staining, we found that the OVA_257–264_–specific TCR expression on DNA origami–based aAPCs–activated T cells (7.89% for aAPC-T3, 12.4% for aAPC-T6, 18.3% for aAPC-T9, and 29.2% for aAPC-T12; Fig. 4, B and C) was much higher than that on naive T cells (1.78%; fig. S18), revealing that those activated T cells were antigen-specific cytotoxic T lymphocytes. Next, the activated splenocytes and target B16-OVA cells (or nontarget B16 cells) were coincubated for 4 hours, and lactate dehydrogenase (LDH) leakage assay was carried out to assess the specific cytolytic activity of activated T cell (*43*). Naive splenocytes had no notable cytolytic activity on B16-OVA and B16 cells (Fig. 4D and fig. S19). In contrast, activated splenocytes presented markedly increased cytotoxicity to B16-OVA cells. These observations further support the importance of pMHC density for efficient T cell activation and targeted tumor cell killing.

### In vivo antitumor activity of optimized DNA origami–based aAPCs-activated T cells

**Fig. 5.**
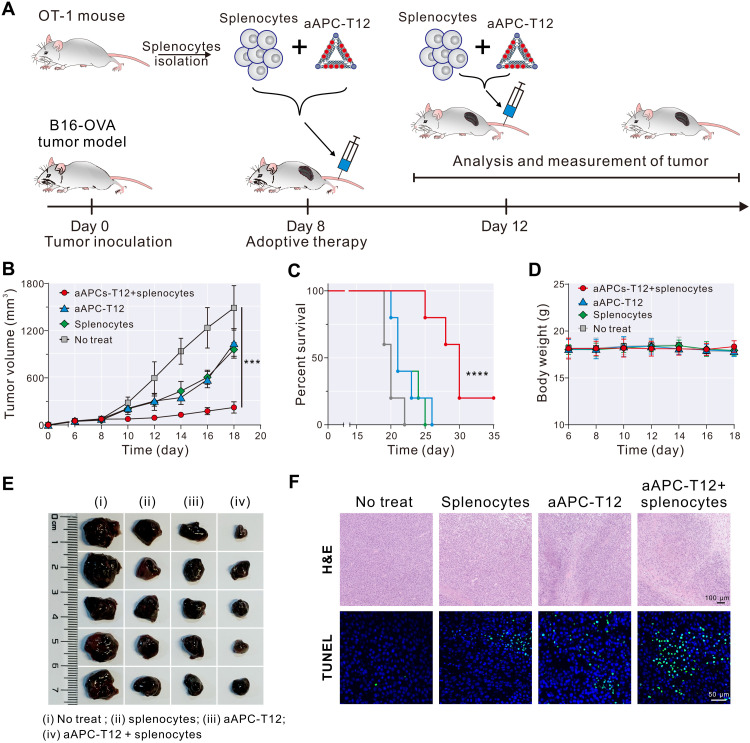
Adoptive immunotherapy with aAPC-T12–expanded T cells delays the tumor growth in a murine melanoma model. (**A**) Work flow diagram depicting the adoptive immunotherapy with aAPC-T12. (**B**) Tumor growth curves of each group (five mice per group). Data are shown as means ± SD (*n* = 5). (**C**) Survival curves of each group. (**D**) Body weights of all mice for each group. Data are shown as means ± SD (*n* = 5). (**E**) Representative tumor images from mice euthanized at day 18 for each group. i, no treat; ii, splenocytes; iii, aAPC-T12; iv, aAPC-T12 + splenocytes. (**F**) Histological observation of (top) hematoxylin and eosin (H&E)–stained tumor tissues after treatment and (bottom) TUNEL-stained tumor tissues after treatment. Two-tailed *t* test was used to calculate *P* values. ***P < 0.001 and ****P < 0.0001.

We next applied the optimized aAPC-T12 for adoptive immunotherapy of the B16-OVA mouse tumor model. The treatment was started by simultaneous intravenous injection of splenocytes extracted from OT-1 mice (2 × 10^7^ per mouse) and aAPC-T12 on the 8th day after tumor implantation (or intravenous injection of only splenocytes or aAPC-T12). Those mice were treated with a total of two injections with a time interval of 4 days (Fig. 5A). In the meantime, the tumor size and mouse survival rate were monitored. Compared with untreated mice, different treatments had noticeable effects on inhibiting the tumor growth (Fig. 5B). In particular, simultaneous injection of splenocytes and aAPC-T12 showed remarkably higher inhibition efficacy toward tumor growth than injection with only splenocytes or only aAPC-T12 (Fig. 5B), indicating the excellent therapeutic response of adoptive immunotherapy using aAPC-T12. The high antitumor activity resulted in greatly prolonged survival time (Fig. 5C). Note that the body weights of the mice had no obvious change during the treatment (Fig. 5D), suggesting the safety of adoptive immunotherapy. Moreover, histologic images using hematoxylin and eosin staining confirmed that a massive cancer cell remission occurred in the tumor tissue after applying simultaneous injection of splenocytes and aAPC-T12, whereas tissue samples from tumors in mice without treatment showed high extent of cytologic polymorphism (Fig. 5F, top). Furthermore, the terminal deoxynucleotidyl transferase–mediated deoxyuridine triphosphate nick end labeling (TUNEL) staining showed that the green fluorescent signal in the nuclei of the aAPC-T12 + splenocytes group was much stronger than other three control groups (Fig. 5D, bottom). These results clearly demonstrated that our constructed DNA origami–based aAPCs (i.e., aAPC-T12) allow for efficient T cell activation and achieve optimal antitumor efficacy in adoptive immunotherapy.

## DISCUSSION

In summary, we have developed DNA origami–based aAPCs with optimized performance in T cell activation for adoptive T cell therapy in vivo. Our constructed DNA origami–based aAPCs present several distinctive advantages for adoptive T cell therapy. First, DNA origami–based aAPCs enable facile and precise control over spatial presentation of activating ligands, enabling the monitoring of individual aAPC-TCR–binding events and spatially correlating to cellular activation. On the above basis, DNA origami–based aAPCs could enable precise regulation of T cell response, in which aAPCs with higher pMHC density present robust binding and avid interaction with T cells, thus triggering more efficient T cell activation. Third, given their good biocompatibility and efficient signal presentation, these nanosized DNA origami–based aAPCs can be administered directly into patients to stimulate CD8^+^ T cell responses in situ, allowing for true off-the-shelf cancer immunotherapy. Collectively, these features are anticipated to make this DNA origami–based aAPCs a powerful tool for adoptive T cell immunotherapy.

## MATERIALS AND METHODS

### Materials and reagents

M13mp18 DNA was purchased from New England Biolabs Inc. (Ipswich, MA, USA). Biotin-labeled pMHC monomer (H-2K^b^, OVA_257–264_) and tetramer (H-2K^b^, OVA_257–264_) were obtained from MBL Biotech Co. Ltd. (Japan), and SA was purchased from Solarbio Science and Technology Co. Ltd. (Beijing, China). CellTrace CFSE cell proliferation kit, IFN-γ enzyme-linked immunosorbent assay (ELISA) kit, and cell culture medium were provided by Thermo Fisher Scientific. Fluorescein isothiocyanate (FITC) anti-mouse CD3 antibody (catalog no. 100204, clone 17A2), BV570 anti-mouse CD8 antibody (catalog no. 100739, clone 53-6.7), and phycoerythrin (PE) anti-mouse CD69 antibody (catalog no. 104507, clone H1.2F3) were purchased from BioLegend (San Diego, CA). Extruder set with Holder/Heating Block (SKU 610000-1Ea) was purchased from Avanti Polar Lipids (Alabaster, AL, USA). All oligonucleotides in this work were synthesized by Sangon Biotech Co. Ltd. (Shanghai, China) with standard desalting, purified with high-performance liquid chromatography, and used without further purification. All other chemicals were obtained from Sigma-Aldrich.

### Synthesis of biotinylated DNA origami

Typically, biotinylated DNA origami was synthesized by mixing M13mp18 with staple strands (including biotinylated DNA and overhang DNA) at a mole ratio of 1: 10 in 50 μl of 1× TAE-Mg^2+^ buffer [40 mM tris, 20 mM acetic acid, 2 mM EDTA, and 12.5 mM magnesium acetate (pH 8.0)], heating from 25° to 95°C for 5 min, and slowly cooling down from 95° to 25°C for 7000 s using a PTC-200 Peltier Thermal Cycler. Next, the product was purified with spin columns (Millipore Amicon Ultra 100 kDa) by centrifugation at 5000*g* for 3 min to remove excess staple strands and repeated three times. Last, the purified biotinylated DNA origami was stored at 4°C for further use. The concentration of biotinylated DNA origami was estimated by measuring its absorption at 260 nm.

### Fabrication of DNA origami–based aAPCs

Before fabrication of DNA origami–based aAPCs, SA-modified DNA origami was assembled by incubating biotinylated DNA origami with SA at a molar ratio of 1:5*n* (*n* indicates the stoichiometric number of SA-binding sites) for 30 min based on SA-biotin interaction. After removing excess SA by ultrafiltration, the produced SA-modified DNA origami was mixed with biotinylated pMHC molecules in 1× TAE buffer at a molar ratio of 1:10*n* and incubated for 30 min. Afterward, the product was purified by ultrafiltration at 5000*g* for 3 min to remove the excess biotinylated pMHC molecules, yielding pMHC-modified DNA origami (denoted as pMHC-T*n*). For the ligation of cDNA (complementary to overhangs at the vertices of DNA origami) onto aCD28, biotin-labeled aCD28 was first incubated with two times excess avidin for 30 min at room temperature, followed by addition of 5′ biotin-labeled cDNA with the same molar amount to that of SA for 30 min of incubation. Then, pMHC-modified DNA origami was incubated with the synthesized aCD28-conjugated cDNA for 1 hour at room temperature. After removing the excess aCD28-conjugated cDNA by ultrafiltration, DNA origami–based aAPCs were yielded and stored at a concentration of 10 nM in 1× TAE buffer at 4°C for further use, and all volumes refer to DNA origami–based aAPCs at this concentration.

### AFM imaging

The fabricated DNA origami–based aAPCs were characterized with AFM (a multimode atomic force microscope, Bruker, USA). For AFM imaging, 5 μl of the diluted samples was directly deposited on the freshly cleaned mica and left to adsorb on the surface for 3 min. Subsequently, the substrate was washed with distilled deionized (DI) water to remove unabsorbed samples and dried with compressed N_2_. Then, the samples were scanned in ScanAsyst Mode (ScanAsyst-Air tips with 0.4 N/m of spring constant), and a resolution of 512 pixels per line with 1-Hz scan rate was chosen for imaging the DNA origami–based aAPCs.

### AGE electrophoresis

The 1% AGE was prepared with 1× TAE-Mg^2+^ buffer, run at 100 V for 90 min in an ice bath, and visualized under ultraviolet light. Last, the band was visualized on a gel imaging system (Tanon Science & Technology Co. Ltd., China).

### Stability study of DNA origami–based aAPCs

To study their structural stability, DNA origami–based aAPCs were incubated in PBS at 37°C. At a preset time (0, 12, 24, 48, 72 hours), AGE analysis was carried out to evaluate the structural integrity of DNA origami–based aAPCs.

### Cell line and animals

Male (or female) C57BL/6 mice and OT-1 TCR transgenic mice (aged 6 to 8 weeks) were provided by Shanghai Model Organisms Biological Technology Co. Ltd. C57BL/6 mice and OT-1 TCR transgenic mice were on a pure C57BL/6 genetic background. All animal studies were approved by the Institutional Animal Care and Use Committee at East China Normal University (protocol no. m20181101). Mouse splenocytes were harvested from C57BL/6 or OT-1 mice. Typically, C57BL/6 or OT-1 mice were euthanized, and the spleen was taken out and placed into a cell culture dish, followed by grinding using a syringe handle. After adding red blood cell lysis buffer for lysis and centrifugation (1000 rpm for 3 min) for collecting the precipitation, the obtained splenocytes were cultured in T cell medium (RPMI 1640 containing 10% FBS and 1% penicillin-streptomycin) at 37°C in a humidified atmosphere with 5% CO_2_.

### T cell isolation

CD8^+^ T cells were isolated from splenocytes and enriched with CD8^+^ T cell isolation kit (BioLegend). The enriched population was stained with FITC anti-CD3, BV570 anti-CD8, and aqua live/dead cell staining and analyzed by fluorescence-activated cell sorting (FACS) for purification. The purity of CD8^+^ T cells was consistently more than 90%. CD8^+^ T cells were resuspended in T cell medium at 37°C in a humidified atmosphere with 5% CO_2_.

### Cytotoxicity evaluation

For cytotoxicity evaluation, the splenocytes extracted from C57 mice were seeded in a 96-well plate at a density of 1 × 10^4^ per well. After culture overnight, the cells were exposed to different concentrations of DNA origami–based aAPCs (5, 10, 15, and 20 nM) and incubated for 12 hours, followed by adding 20 μl of the 3-(4,5-dimethylthiazol-2-yl)-2,5-diphenyltetrazolium bromide (5 mg/ml) into each well for incubation of another 4 hours. After removing the supernatant, cells were mixed with 150 μl of dimethyl sulfoxide, and their absorbance was recorded at a test wavelength of 570 nm and a reference wavelength of 630 nm by a microplate reader (Varioskan LUX, USA).

### Measurement of Ca^2+^ signal

For assessment of early T cell activation, Ca^2+^ signals were measured. Typically, T cells were resuspended in PBS after incubation with 10 μl of DNA origami–based aAPCs for 1 hour and cultured with 5 μM Fluo-4 AM in the dark at 37°C. After incubation for another 1 hour, cells were washed three times with PBS and kept in the dark for 20 min to allow de-esterification of the dye. Then, Ca^2+^ imaging was conducted at 37°C on an inverted fluorescence microscope (Nikon Eclipse Ti), and its fluorescence signal was measured by fluorescence spectrometer (F-7000).

### Analysis of CD69 expression

For assessment of T cell activation, CD69 expression of CD8^+^ T cells was analyzed. Freshly isolated CD8^+^ T cells (2 × 10^5^ per well) were first incubated with 10 μl of DNA origami–based aAPCs in T cell medium for 16 hours. After being washed with PBS, harvested cells were stained with anti-mouse CD69-PE in FACS buffer (1× PBS, 0.1% heat-inactivated FBS, and 0.1% NaN_3_) for 30 min on ice. Afterward, the staining cells were washed with PBS, resuspended in 100 μl of FACS buffer, and analyzed using flow cytometry.

### IFN-γ assay

To study secretion of IFN-γ cytokines by activated T cells, IFN-γ assay was performed. Freshly isolated CD8^+^ T cells (2 × 10^5^ per well) were first incubated with 10 μl of DNA origami–based aAPCs in T cell medium for 16 hours. Then, IFN-γ concentration in culture supernatants was detected by mouse IFN-γ ELISA Ready-Set-Go kit (eBioscience, Frankfurt, German) according to the manufacturer’s instructions.

### Cell proliferation

Before detecting CD8^+^ T cell proliferation by flow cytometry, CD8^+^ T cells were stained with fluorescent dye 5-(and -6)-CFSE (Invitrogen, Life Technologies GmbH, Darmstadt, Germany). First, freshly isolated CD8^+^ T cells (2 × 10^6^ cells/ml) in 1 ml of RPMI 1640 were stained with 8 μM CFSE for 10 min at room temperature in the dark. Then, CFSE-stained CD8^+^ T cells were collected by centrifugation (1000 rpm for 5 min) and resuspended in T cell medium. Next, the suspended CD8^+^ T cells (2 × 10^5^ cells/ml) were incubated with 10 μl of DNA origami–based aAPCs in T cell medium supplemented with recombinant interleukin-2 (30 U/ml). After 3 days of incubation, CD8^+^ T cells were collected by centrifugation (1000 rpm for 3 min) and stained with aqua live/dead cell stain in FACS buffer. Last, cells were analyzed on the flow cytometer. The cell proliferation was analyzed by the dilution of CFSE fluorescence.

### Bright field imaging of T cells

Bright-field images were acquired on an inverted microscope (Nikon Eclipse Ti) after incubation of T cells with 10 μl of DNA origami–based aAPCs for 3 days, and collected images were further processed with ImageJ.

### Preparation of SLB

SLBs were prepared by spreading small unilamellar vesicles (SUVs) on a cover glass. SUVs were first synthesized as follows: A desired volume of chloroform solution of dioleoylphosphatidylcholine (DOPC) was added in a 25-ml round-bottomed flask and evaporated under a stream of N_2_. Then, the dried mixture was dispersed in DI water and sonicated for 1 hour. After that, the solution was extruded 40 times through a polycarbonate membrane with a pore diameter of 100 nm, and the prepared SUV was kept at 4°C and used within 1 week.

To prepare SLBs, cover glass was used as the sample chamber for supporting SLBs. The used cover glass was cleaned with plasma cleaner (PDC-MG, Ming Heng) for 1 min, immersed in 1% hydrofluoric acid for 30 s, and thoroughly washed with DI water. After drying with N_2_, a quadrate chip fence was stuck to the cover glass to construct a sample chamber, and 50 μl of SUV solution was added into the sample chamber. After 30 min of incubation at 25°C, excess unfused SUVs were removed by thoroughly rinsing with PBS, and their fluidity was confirmed with FRAP experiments.

### FRAP experiment

To determine the diffusion coefficient (*D*) of SLBs [composed of DOPC and Dio probe (Beyotime, China) with a mole ratio of 99: 1], FRAP experiment was carried out. In this work, a circular bleach spot with a radius of 15 μm was used as the model for data collection. A 20- to 30-min video was acquired for recording the recovery after bleaching (at 488-nm laser). The FRAP curve was fitted to a one-phase association fit model using Eq. 2

$$F(t)=a+b(1-e^{-\lambda t})$$ (2)

Here, the characteristic diffusion time (τ_1/2_) can be calculated using Eq. 3

$$\tau_{1/2}=\frac{\text{In}2}{\lambda }$$ (3)

The diffusion coefficient (*D*) was calculated using Eq. 4

$$D=\frac{0.224W^{2}}{\tau_{1/2}}$$ (4)

where *W* is the radius of the photobleached spot.

### SLBs assembly and TIRF imaging

To monitor the DNA origami–based aAPC-TCR–binding events, DNA origami–based aAPCs were tethered on SLBs via DNA hybridization. Briefly, 50 μl of cholesterol-modified single-stranded DNA (chol-DNA; 200 nM) in PBS was dropcasted on the as-prepared SLBs. After 30 min of incubation at 4°C, excess chol-DNA strands were washed out with PBS. Before imaging, the SLBs was incubated with appropriate concentrations of DNA origami–based aAPCs and ICAM-1 (~100 nM) in live T cell–imaging buffer (LCB; 1 mM CaCl_2_, 2 mM MgCl_2_, 20 mM Hepes, 137 mM NaCl, 5 mM KCl, 0.7 mM Na_2_HPO_4_, 6 mM d-glucose, and 1% (w/v) bovine serum albumin] for 30 min at room temperature. After incubation, the SLBs was rinsed with LCB. Then, T cells resuspended in LCB were introduced to the SLBs. All imaging was done at room temperature.

TIRF imaging was conducted on a commercial TIRF microscope (N-storm, Nikon) with a 100× objective lens (numerical aperture, 1.49) and electron multiplying charge-coupled device camera (Andor, iXon 3). After T cell seeding to the SLBs, the imaging region was monitored upon exciting with the 488-nm laser. TIRF images were collected for 90 s with an about 560-ms time resolution using a low laser illumination intensity (0.1 mW/cm^2^).

### Imaging analysis

TIRF images were processed using ImageJ software. The plugin “TrackMate” in ImageJ was used for tracking individual AF488-labeled DNA origami–based aAPCs. The mean dwell time (<τ_off_>) was calculated using rates extracted from a mathematical fit of the experimental data and calibrated for photobleaching based on Eqs. 5 and 6 (*41*, *44*)

$$f(\tau_{\mathrm{obs}})=e^{-(<\tau_{\text{bl}}{>}^{-1}+<\tau_{\text{off}}{>}^{-1})\tau_{\text{obs}}}$$ (5)

$$<\tau_{\text{obs}}{>}^{-1}=<\tau_{\text{off}}{>}^{-1}+<\tau_{\text{bl}}{>}^{-1}$$ (6)

where <τ_bl_>^−1^ is the photobleaching rate (*k*_off_), <τ_off_>^−1^ is the unbinding rate (*k*_off_), and <τ_obs_> is the observed mean dwell time in the experiment. By measuring both <τ_bl_> and < τ_obs_>, <τ_off_> can be determined when <τ_obs_> ≤ <τ_bl_> (*44*). The bleaching times <τ_bl_> of DNA origami–based aAPCs are 76.1 ± 11.9, 76.8 ± 12.6, 76.5 ± 13.7, and 76.0 ± 13.1 s for aAPC-T3, aAPC-T6, aAPC-T9, and aAPC-T12, respectively. The mean dwell times (<τ_off_>) of DNA origami–based aAPCs are 9.9 ± 0.8, 10.9 ± 0.4, 11.0 ± 0.2, and 12.1 ± 0.3 s for aAPC-T3, aAPC-T6, aAPC-T9, and aAPC-T12, respectively.

### MSD analysis

The diffusion coefficient (*D*) of DNA origami–based aAPCs was calculated on the basis of their MSD. For each particle and every time interval *t*, the MSD was calculated using Eq. 7 (*45*)

$$\text{MSD}(t)=\text{MSD}[n({\mathrm{fr}}_{\text{time}})]=1/N\sum_{i=1}^{N}[{\left(x_{i+n}-x_{i}\right)}^{2}+{\left(y_{i+n}-y_{i}\right)}^{2}]$$ (7)

where *N* is the number steps, *n* is the step size in frames ranging from 1 to ^1^/_4_ of the available frames, *fr*_time_ is the time between two consecutive frames, and *x* and *y* correspond the particle position at the frame. The MSD analysis was implemented in MATLAB.

### In vitro cell killing assay

To assess T cell cytolytic activity, in vitro cell killing assay was carried out. Before conducting assay, splenocytes from C57 mouse were extracted as described above. Then, splenocytes (2 × 10^6^ cells/ml) were incubated with 10 μl of DNA origami–based aAPCs in T cell medium for 3 days, followed by washing with PBS. Then, the stimulated splenocytes were cocultured with B16-OVA target cells or B16 cells (8 × 10^3^ cells per well) with a cell ratio of 10:1 in 96-well plates for 4 hours. According to the vendor’s instruction manual, LDH leakage was detected with LDH detection kit (Nanjing Jiancheng Bioengineering Institute) in the collected culture suspensions. The LDH leakage indicates the level of specific lysis of target cells by the activated T cells. Percentage of specific lysis was calculated as follows: 100 × (experimental − T cell spontaneous − target cell spontaneous)/(target cell maximum − target cell spontaneous).

### In vivo tumor–adoptive immunotherapy

For tumor adoptive immunotherapy, B16-OVA tumor model on C57 mice was first established. C57 tumors were inoculated by subcutaneous injection of 50 μl of PBS containing 1 × 10^6^ cells into the back of each mouse. After inoculation for 8 days, three groups (five mice per group) of treatments were conducted, including simultaneous injection of splenocytes of OT-1 mouse (2 × 10^7^) and aAPC-T12, only injection of splenocytes of OT-1 mouse (2 × 10^7^), and only injection of aAPC-T12 two times with a 4-day interval. Untreated group (five mice) was also performed at the same time. Mice were randomly divided into each group. Tumor volumes and survival of different groups of mice were monitored.

### Statistical analysis

All the experiments were independently repeated at least three times. Two-tailed *t* test was used to calculate *P* values. ***P < 0.001 and ****P < 0.0001.

## Notes

View/request a protocol for this paper from *Bio-protocol*.
