# Supplementary material for: DNA origami–based artificial antigen-presenting cells for adoptive T cell therapy
Source: Sci Adv. 2022 Dec 2;8(48):eadd1106. doi: 10.1126/sciadv.add1106 (PMC10936057; doi:10.1126/sciadv.add1106)
Supplement: Supplementary file 1 — Figs. S1 to S19 Tables S1 and S2 [file sciadv.add1106_sm.pdf]

Science Advances
AAAS

Supplementary Materials for

DNA origami–based artificial antigen-presenting cells for adoptive T cell therapy

Yueyang Sun *et al.*

Corresponding author: Li Li, lli@chem.ecnu.edu.cn; Hao Pei, peihao@chem.ecnu.edu.cn

Sci. Adv. **8**, eadd1106 (2022)
DOI: 10.1126/sciadv.add1106

This PDF file includes:

Figs. S1 to S19
Tables S1 and S2

Figures and Tables

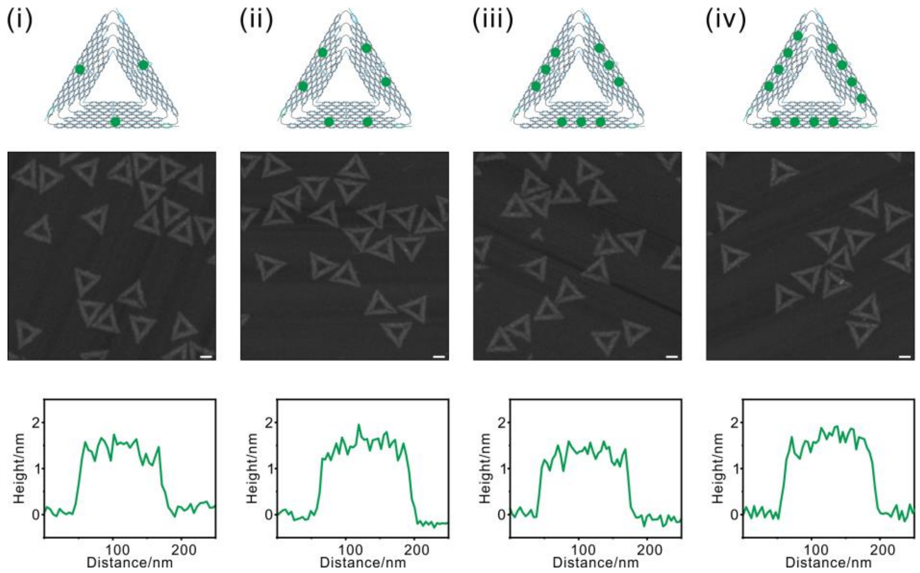

Fig. S1. Synthesis and characterization of biotinylated DNA origamis. Scheme and AFM images of biotinylated DNA origamis and their corresponding cross-section height analysis results. Biotin (SA binding site) was represented as the green dot. Scale bar: 50 nm.

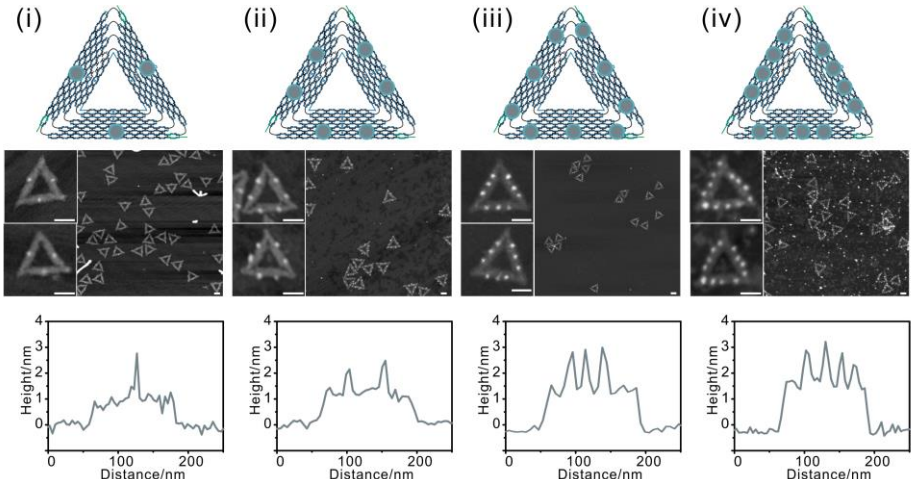

Fig. S2. Synthesis and characterization of SA-modified DNA origamis. Scheme and AFM images of SA-modified DNA origamis and their corresponding cross-section height analysis results. SA was represented as the gray dot. Scale bar: 50 nm.

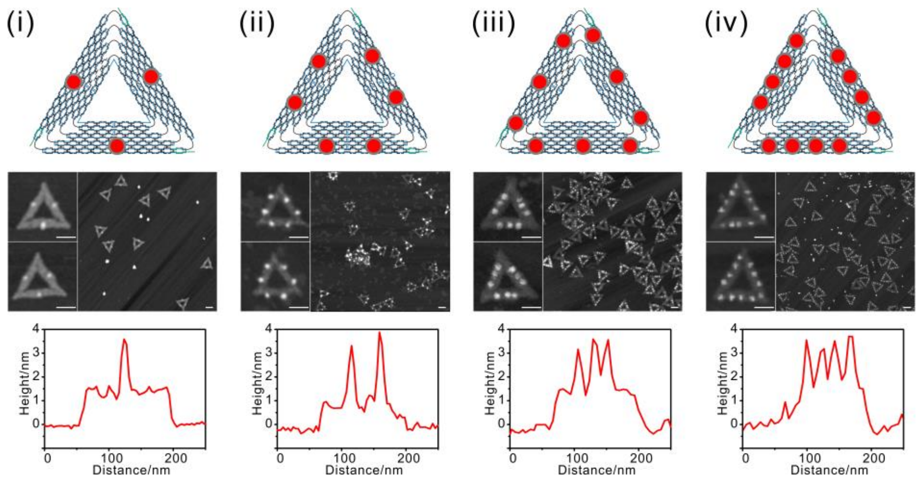

Fig. S3. Synthesis and characterization of pMHC-modified DNA origami. Scheme and AFM images of pMHC-modified DNA origami (including pMHC-T3, pMHC-T6, pMHC-T9, and pMHC-T12) and their corresponding cross-section height analysis results. pMHC was represented as the red dot. Scale bar: 50 nm.

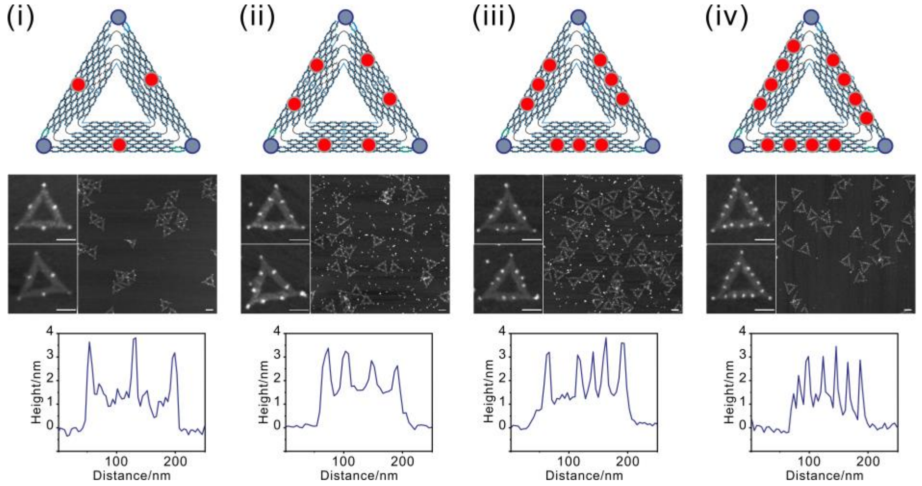

Fig. S4. Synthesis and characterization of DNA origami-based aAPCs. Scheme and AFM images of four types of DNA origami-based aAPCs, including aAPC-T3, aAPC-T6, aAPC-T9, aAPC-T12, and their corresponding cross-section height analysis results. pMHC was represented as the red dot. aCD28 was represented as the blue dot. Scale bar: 50 nm.

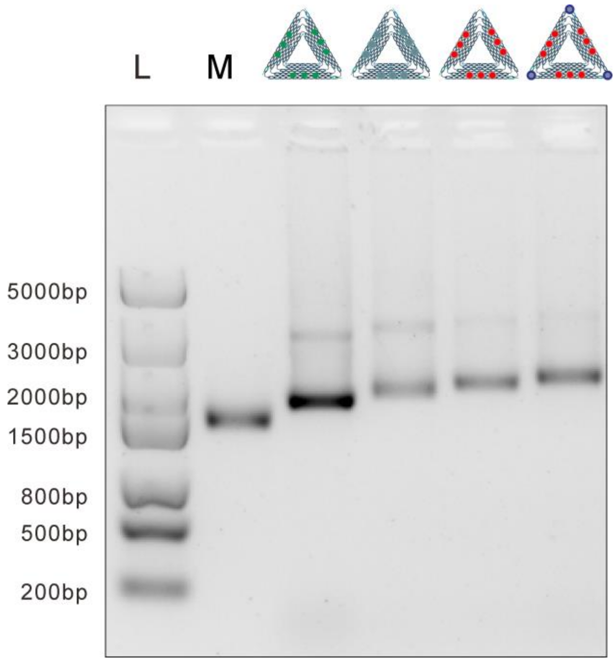

Fig. S5. AGE analysis of formation of DNA origami-based aAPCs (i.e., aAPC-T9). First lane: DNA ladder (L), second lane: M13mp18 scaffold (M), third lane: biotinylated DNA origami, fourth lane: SA-modified DNA origami, fifth lane: pMHC-modified DNA origami, sixth lane: aAPC-T9. All DNA origami-related products were analyzed after purification.

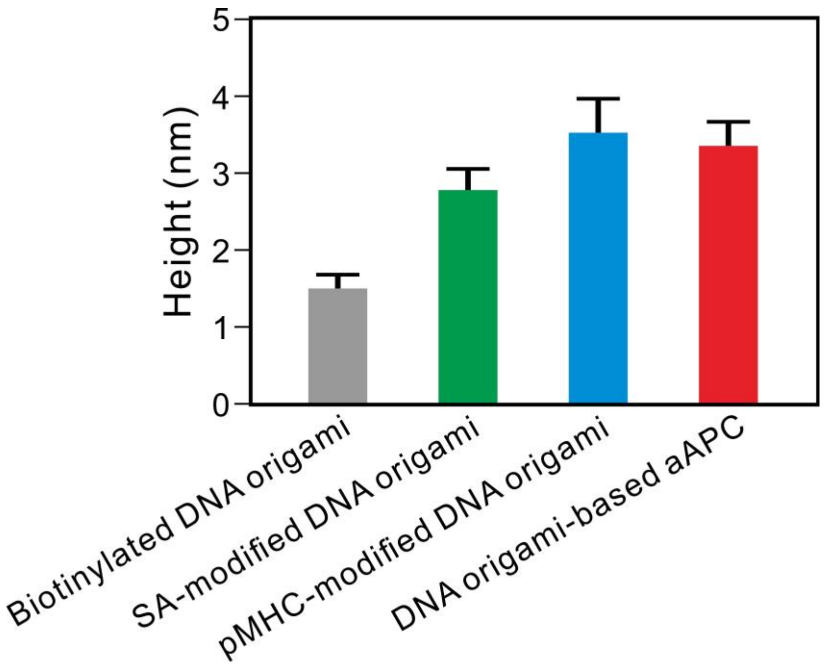

Fig. S6. Cross-section height analysis of formation of DNA origami-based aAPCs. Cross-section height analysis of biotinylated DNA origamis, SA-modified DNA origamis, pMHC-modified DNA origamis, DNA origami-based aAPCs. Data are shown as means ± s.d.

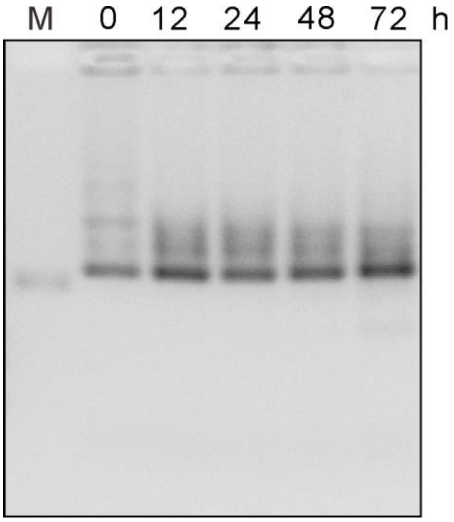

Fig. S7. Stability analysis of DNA origami-based aAPCs in PBS. The long-term stability analysis of DNA origami-based aAPCs in PBS for different times. (M: M13mp18 scaffold)

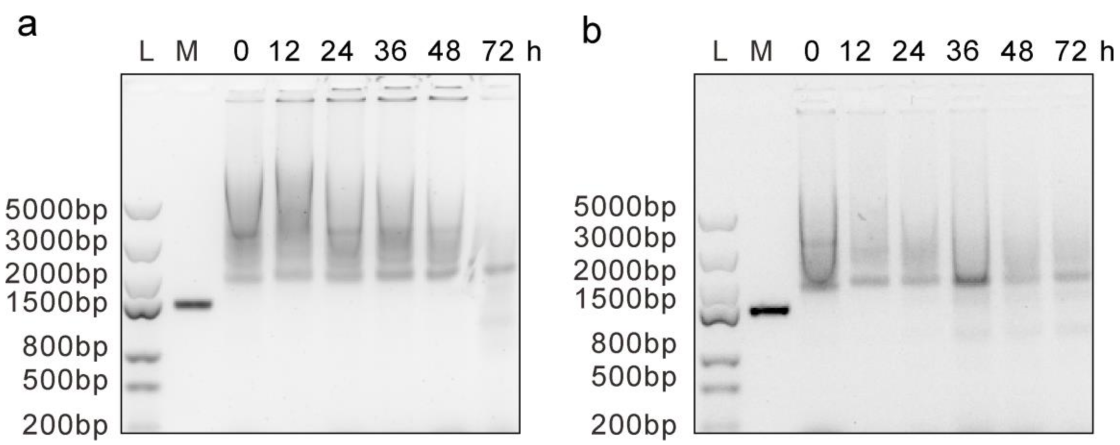

Fig. S8. Stability analysis of DNA origami-based aAPCs in 10% FBS. The long-term stability analysis of DNA origami-based aAPCs (i.e., aAPC-T9) in 10% FBS for different temperatures at 25 °C (**a**) and 37 °C (**b**). (L: DNA ladder, M: M13mp18 scaffold)

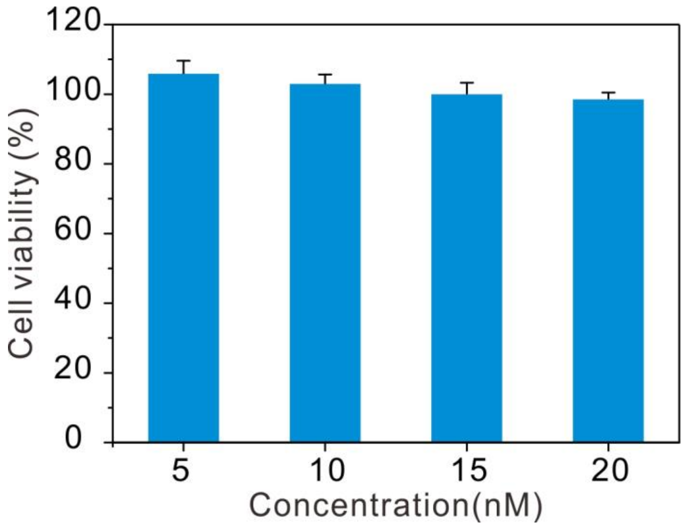

Fig. S9. Biocompatibility analysis of DNA origami-based aAPCs. Cytotoxicity assessment of different concentrations of DNA origami-based aAPCs (i.e., aAPC-T9) to spleen cells after 12 h incubation. Data are shown as means ± s.d. (n = 5).

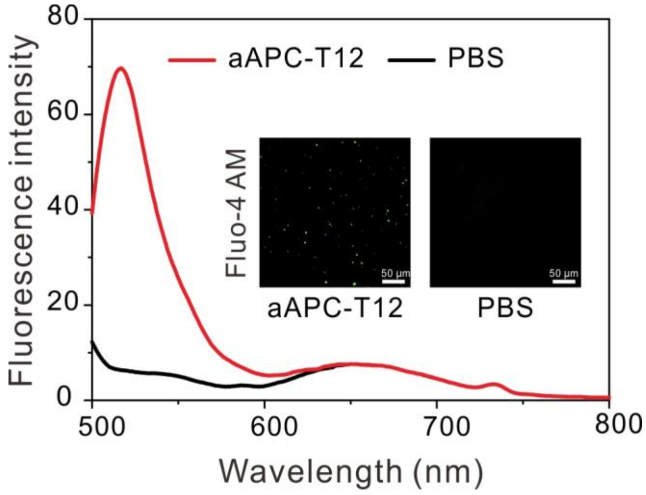

Fig. S10. Ca^{2+} signaling analysis after stimulation. Ca^{2+} signaling measurements by fluorescence spectrometry and fluorescence microscopy after stimulation of T cells with aAPC-T12 and PBS for 1 h.

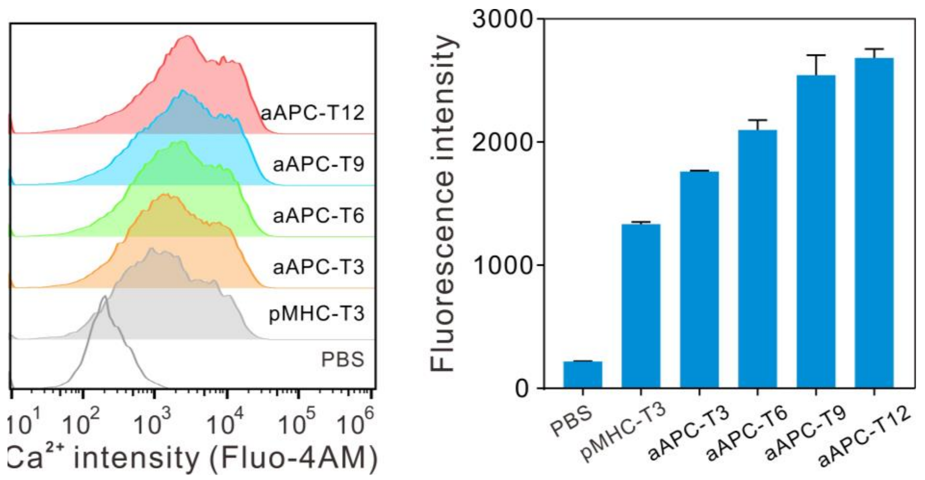

Fig. S11. Ca^{2+} signaling analysis after stimulation by DNA origami-based aAPCs. Ca^{2+} signaling measurements with flow cytometry after stimulation of T cells with DNA origami-based aAPCs (aAPC-T3, aAPC-T6, aAPC-T9, aAPC-T12), pMHC-T3, and PBS for 1 h. Data are shown as means ± s.d. (n = 3)

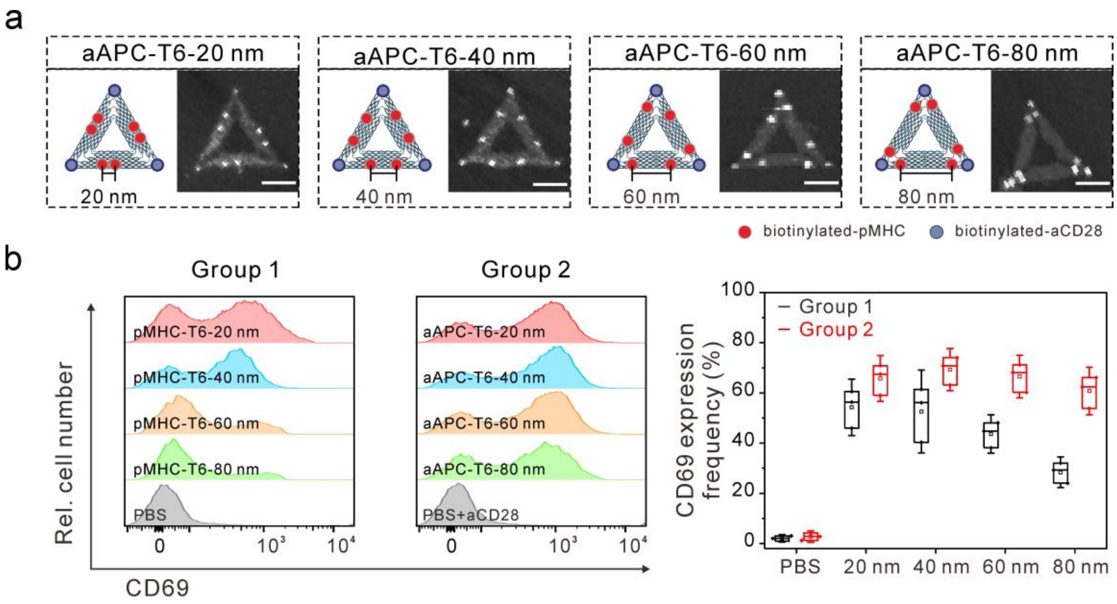

Fig. S12. Synthesis and performance of DNA origami-based aAPCs with the same pMHC copy number and different inter-pMHC spacing. (**a**) Scheme and AFM images of DNA origami-based aAPCs with the same pMHC copy number and different inter-pMHC spacing, including aAPC-T6-20 nm, aAPC-T6-40 nm, aAPC-T6-60 nm, aAPC-T6-80 nm. Scale bar: 50 nm. (**b**) Flow cytometry analysis of CD69 expression of T cells after stimulation by DNA origami-based aAPCs. Data are shown as means ± s.d. (n = 3).

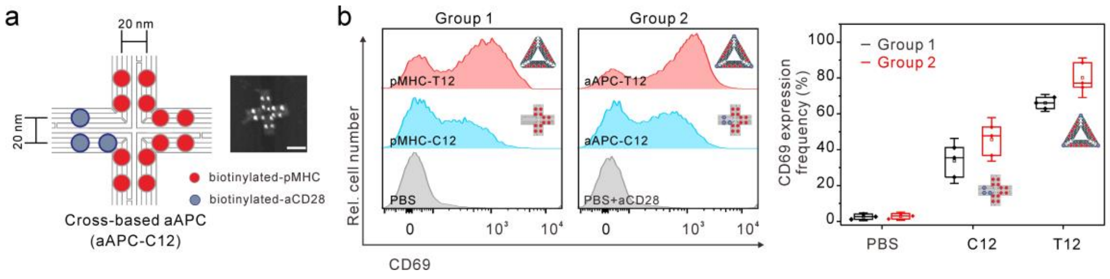

Fig. S13. Synthesis and performance of cross-based aAPC. (**a**) Scheme and AFM image of DNA origami-based aAPC assembled by a cross DNA origami platform (denoted as cross-based aAPC, aAPC-C12). The ligands of pMHC and aCD28 were on the same side of the cross origami, and the copy numbers of pMHC and aCD28 of cross-based aAPC were the same as those of aAPC-T12 and the inter-pMHC spacing at each edge of the cross origami was 20 nm. Scale bar: 50 nm. (**b**) Flow cytometry analysis of CD69 expression of T cells after stimulation by aAPC-C12 and aAPC-T12. Data are shown as means ± s.d. (n = 3)

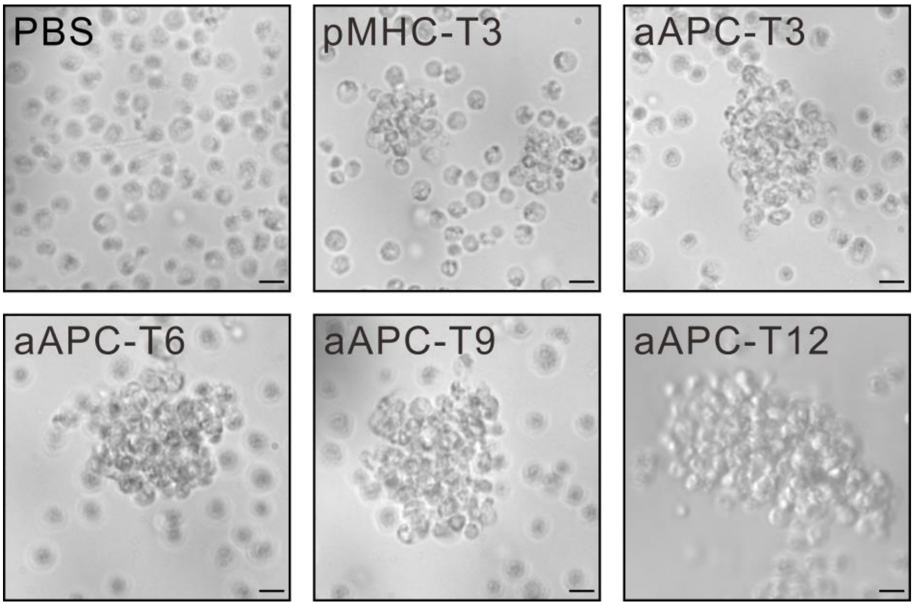

Fig. S14. Long-term T cell survival analysis. Representative brightfield microscopy images of clusters of activated CD8$^+$ T cells after culturing for 3 days with PBS, pMHC-T3, DNA origami-based aAPCs (aAPC-T3, aAPC-T6, aAPC-T9, aAPC-T12). Scale bar: 10 μm.

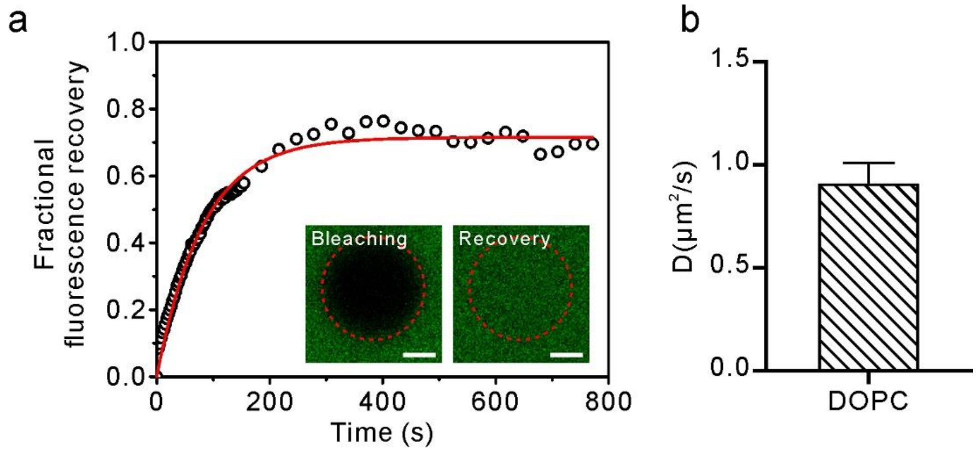

Fig. S15. Fluidity and integrality analysis of SLBs. (**a**) FRAP curve of a supported bilayer composed of DOPC lipids. Inset: fluorescence images of region of interest (in dash line) after initial photobleaching and fluorescence recovery. Scale bar: 10 μm. (**b**) The diffusion coefficient of DOPC lipid bilayer (0.92 ± 0.12 μm2/s). Data are shown as means ± s.d. (n = 3).

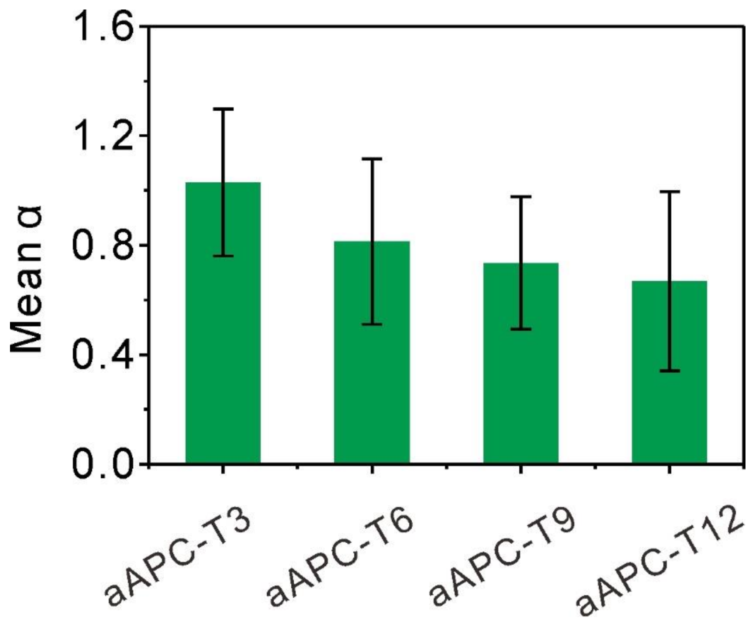

Fig. S16. Mean α of DNA origami-based aAPCs. Mean α of DNA origami-based aAPCs obtained from 25 trajectories in three independent experiments. Mean α: 1.029 ± 0.269, 0.813 ± 0.302, 0.736 ± 0.242, and 0.669 ± 0.328 for aAPC-T3, aAPC-T6, aAPC-T9, and aAPC-T12, respectively. Data are shown as means ± s.d. ($n = 3$).

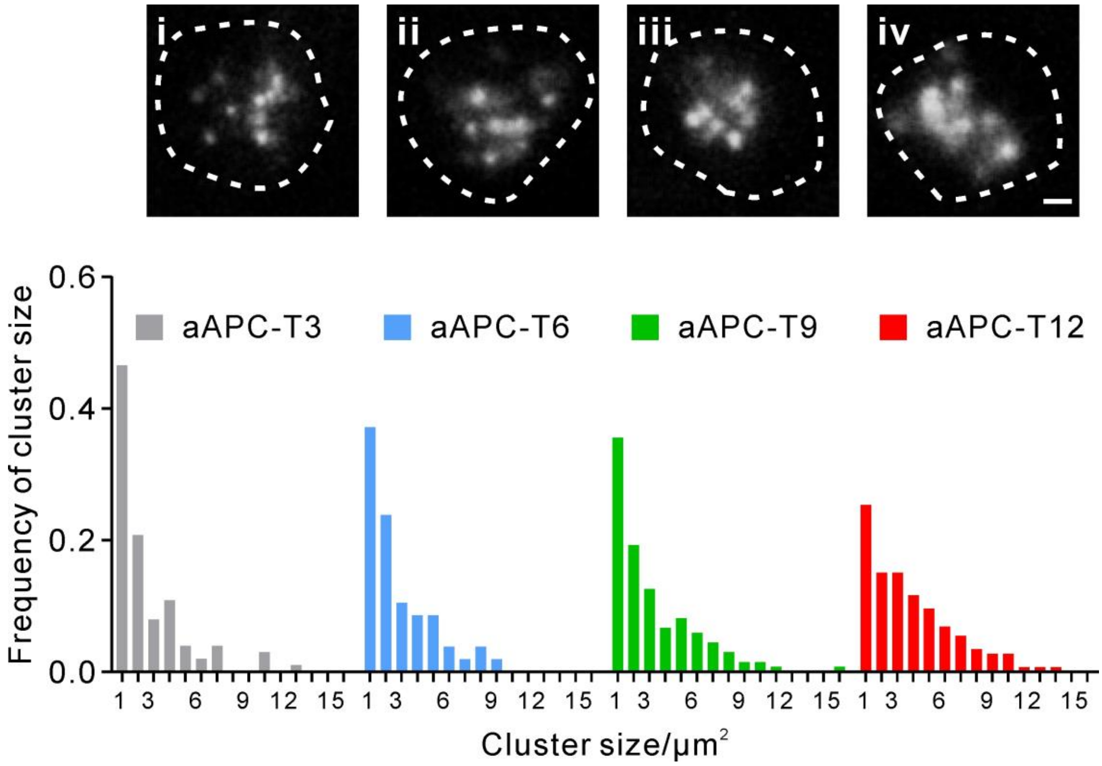

Fig. S17. Size analysis of DNA origami-based aAPCs-TCR complexes clustering. Representative TIRF images for DNA origami-based aAPCs-TCR complexes clustering and size distributions of aAPCs-TCR complexes clusters at the T-cell-SLB interface. Images were recorded 30 min after T cell seeding. Data of size distributions of aAPCs-TCR complexes clusters are from 100 to 150 regions from 30 to 50 cells per condition. Data are from three independent experiments. Scale bar: 2 μm. i: aAPC-T3, ii: aAPC-T6, iii: aAPC-T9, iv: aAPC-T12.

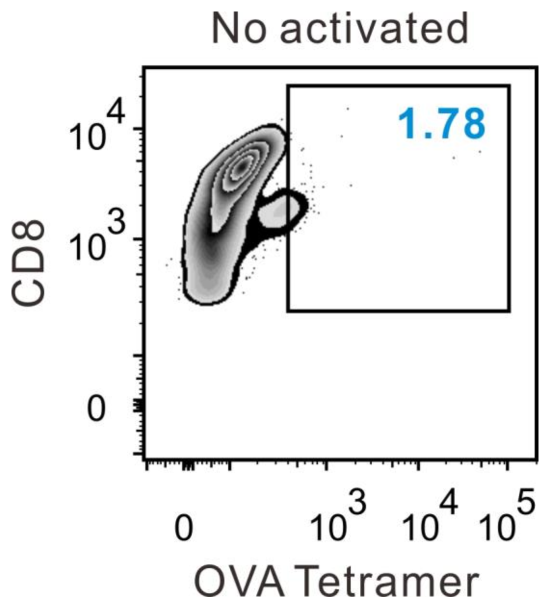

Fig. S18. Tetramer staining in Naive splenocytes. Flow cytometry analysis of $OVA_{257-264}$-specific $CD8^+$ T cell populations in the splenocytes stained by tetramer.

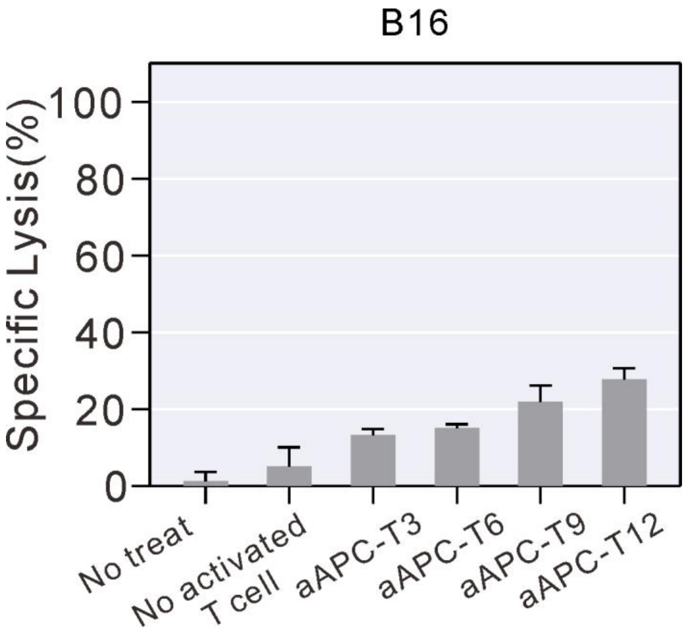

Fig. S19. Cytotoxic activity towards B16 cells. Cytotoxic activity of splenocytes stimulated by DNA origami-based aAPCs towards B16 cells. Data are shown as means ± s.d. (n = 3).

Table S1. The sequences of oligonucleotides for the triangular DNA origami used in this work

Name	The sequences (from 5′ to 3′)
A01	CGGGGTTTCCTCAAGAGAAGGATTTTGAATTA
A02	AGCGTCATGTCTCTGAATTTACCGACTACCTT
A03	TTCATAATCCCCTTATTAGCGTTTTTCTTACC
A04	ATGGTTTATGTCACAATCAATAGATATTAAAC
Biotin-A04	Biotin-TTTTATGGTTTATGTCACAATCAATAGATATTAAAC
A05	TTTGATGATTAAGAGGCTGAGACTTGCTCAGTACCAGGCG
A05-Poly(T)$_{12}$	TTTGATGATTAAGAGGCTGAGACTTGCTCAGTACCAGGCG(T)$_{12}$
AF488-cDNA	AF488-Poly(A)$_{12}$
A06	CCGGAACCCAGAATGGAAAGCGCAACATGGCT
A07	AAAGACAACATTTTCGGTCATAGCCAAAATCA
A08	GACGGGAGAATTAACTCGGAATAAGTTTATTTCCAGCGCC
A09	GATAAGTGCCGTCGAGCTGAAACATGAAAGTATACAGGAG
A10	TGTACTGGAAATCCTCATTAAAGCAGAGCCAC
A11	CACCGGAAAGCGCGTTTTCATCGGAAGGGCGA
A12	CATTCAACAAACGCAAAGACACCAGAACACCCTGAACAAA
A13	TTTAACGGTTCGGAACCTATTATTAGGGTTGATATAAGTA
A14	CTCAGAGCATATTCACAAACAAATTAATAAGT
A15	GGAGGGAATTTAGCGTCAGACTGTCCGCCTCC
A16	GTCAGAGGGTAATTGATGGCAACATATAAAAGCGATTGAG
A17	TAGCCCGGAATAGGTGAATGCCCCCTGCCTATGGTCAGTG
A18	CCTTGAGTCAGACGATTGGCCTTGCGCCACCC
A19	TCAGAACCCAGAATCAAGTTTGCCGGTAAATA
A19-overhangs2	TCAGAACCCAGAATCAAGTTTGCCGGTAAATATTTTTTTTCTCTGGTTAACGTGT CTGGGC
Chol-cDNA2	Chol-TTTTTTTTTTTTTTTTTTTTGCCCAGACACGTTAACCAGAG
A20	GACGGAAATACATACATAAAGGGCGCTAATATCAGAGA
Biotin-A20	Biotin-TTTTTTGACGGAAATACATACATAAAGGGCGCTAATATCAGAGA
A21	CAGAGCCAGGAGGTTGAGGCAGGTAACAGTGCCCG
A22	ATTAAAGGCCGTAATCAGTAGCGAGCCACCCT
A23	GATAACCCACAAGAATGTTAGCAAACGTAGAAAATTATTC
A24	GCCGCCAGCATTGACACCACCCTC
A25	AGAGCCGCACCATCGATAGCAGCATGAATTAT
A26	CACCGTCACCTTATTACGCAGTATTGAGTTAAGCCCAATA
A27	AGCCATTTAAACGTCACCAATGAACACCAGAACCA
A28	ATAAGAGCAAGAAACATGGCATGATTAAGACTCCGACTTG
A29	CCATTAGCAAGGCCGGGGGAATTA
A30	GAGCCAGCGAATACCCAAAAGAACATGAAATAGCAATAGC
Biotin-A30	Biotin-TTTTGAGCCAGCGAATACCCAAAAGAACATGAAATAGCAATAGC
A31	TATCTTACCGAAGCCCAAACGCAATAATAACGAAAATCACCAG
A32	CAGAAGGAAACCGAGGTTTTTAAGAAAAGTAAGCAGATAGCCG
A32-overhang1	CAGAAGGAAACCGAGGTTTTTAAGAAAAGTAAGCAGATAGCCGGTCAGTCAG TCAGTCAGTCA

Biotin-cDNA1	Biotin-TTTTTTTTTGACTGACTGACTGACTGA
A33	CCTTTTTTCATTTAACAATTTCATAGGATTAG
A34	TTTAACCTATCATAGGTCTGAGAGTTCCAGTA
A35	AGTATAAAATATGCGTTATACAAAGCCATCTT
A36	CAAGTACCTCATTCCAAGAACGGGAAATTCAT
A37	AGAGAATAACATAAAAACAGGGAAGCGCATTA
A38	AAAACAAAATTAATTAAATGGAAACAGTACATTAGTGAAT
A39	TTATCAAACCGGCTTAGGTTGGGTAAGCCTGT
A40	TTAGTATCGCCAACGCTCAACAGTCGGCTGTC
A41	TTTCCTTAGCACTCATCGAGAACAATAGCAGCCTTTACAG
A42	AGAGTCAAAAATCAATATATGTGATGAAACAAACATCAAG
A43	ACTAGAAATATATAACTATATGTACGCTGAGA
A44	TCAATAATAGGGCTTAATTGAGAATCATAATT
A45	AACGTCAAAAATGAAAAGCAAGCCGTTTTTATGAAACCAA
A46	GAGCAAAAGAAGATGAGTGAATAACCTTGCTTATAGCTTA
A47	GATTAAGAAATGCTGATGCAAATCAGAATAAA
A48	CACCGGAATCGCCATATTTAACAAAATTTACG
A48-overhangs2	CACCGGAATCGCCATATTTAACAAAATTTACGTTTTTTTTCTCTGGTTAACGTGT CTGGGC
A49	AGCATGTATTTCATCGTAGGAATCAAACGATTTTTTGTTT
Biotin-A49	Biotin-TTTTAGCATGTATTTCATCGTAGGAATCAAACGATTTTTTGTTT
A50	ACATAGCGCTGTAAATCGTCGCTATTCATTTCAATTACCT
A51	GTTAAATACAATCGCAAGACAAAGCCTTGAAA
A52	CCCATCCTCGCCAACATGTAATTTAATAAGGC
A53	TCCCAATCCAAATAAGATTACCGCGCCCAATAAATAATAT
A54	TCCCTTAGAATAACGCGAGAAAACTTTTACCGACC
A55	GTGTGATAAGGCAGAGGCATTTTCAGTCCTGA
A56	ACAAGAAAGCAAGCAAATCAGATAACAGCCATATTATTTA
A57	GTTTGAAATTCAAATATATTTTAG
A58	AATAGATAGAGCCAGTAATAAGAGATTTAATG
A59	GCCAGTTACAAAATAATAGAAGGCTTATCCGGTTATCAAC
A60	TTCTGACCTAAAATATAAAGTACCGACTGCAGAAC
A61	GCGCCTGTTATTCTAAGAACGCGATTCCAGAGCCTAATTT
Biotin-A61	Biotin-TTTTGCGCCTGTTATTCTAAGAACGCGATTCCAGAGCCTAATTT
A62	TCAGCTAAAAAAGGTAAAGTAATT
A63	ACGCTAACGAGCGTCTGGCGTTTTAGCGAACCCAACATGT
A64	ACGACAATAAATCCCGACTTGCGGGAGATCCTGAATCTTACCA
A65	TGCTATTTTGCACCCAGCTACAATTTTGTTTTGAAGCCTTAAA
B01	TCATATGTGTAATCGTAAAACTAGTCATTTTC
B02	GTGAGAAAATGTGTAGGTAAAGATACAACTTT
B03	GGCATCAAATTTGGGGCGCGAGCTAGTTAAAG
B04	CGAGCTAAGACTTCAAATATCGGGAACGAG
Biotin-B04	Biotin-TTTTTTCGAGCTAAGACTTCAAATATCGGGAACGAG

B05	ACAGTCAAAGAGAATCGATGAACGACCCCGGTTGATAATC
B05-Poly(T)$_{12}$	ACAGTCAAAGAGAATCGATGAACGACCCCGGTTGATAATC(T)$_{12}$
B06	ATAGTAGTATGCAATGCCTGAGTAGGCCGGAG
B07	AACCAGACGTTTAGCTATATTTTCTTCTACTA
B08	GAATACCACATTCAACTTAAGAGGAAGCCCGATCAAAGCG
B09	AGAAAAGCCCCAAAAAGAGTCTGGAGCAAACAATCACCAT
B10	CAATATGACCCTCATATATTTTAAAGCATTAA
B11	CATCCAATAAATGGTCAATAACCTCGGAAGCA
B12	AACTCCAAGATTGCATCAAAAAGATAATGCAGATACATAA
B13	CGTTCTAGTCAGGTCATTGCCTGACAGGAAGATTGTATAA
B14	CAGGCAAGATAAAAATTTTTAGAATATTCAAC
B15	GATTAGAGATTAGATACATTTCGCAAATCATA
B16	CGCCAAAAGGAATTACAGTCAGAAGCAAAGCGCAGGTCAG
B17	GCAAATATTTAAATTGAGATCTACAAAGGCTACTGATAAA
B18	TTAATGCCTTATTTCAACGCAAGGGCAAAGAA
B19	TTAGCAAATAGATTTAGTTTGACCAGTACCTT
B19-overhangs2	TTAGCAAATAGATTTAGTTTGACCAGTACCTTTTTTTTTTTCTCTGGTTAACGTGT CTGGGC
B20	AATTGCTTTACCCTGACTATTATGAGGCATAGTAAGAGC
Biotin-B20	Biotin-TTTTTAATTGCTTTACCCTGACTATTATGAGGCATAGTAAGAGC
B21	ATAAAGCCTTTGCGGGAGAAGCCTGGAGAGGGTAG
B22	TAAGAGGTCAATTCTGCGAACGAGATTAAGCA
B23	AACACTATCATAACCCATCAAAAATCAGGTCTCCTTTTGA
B24	ATGACCCTGTAATACTTCAGAGCA
B25	TAAAGCTATATAACAGTTGATTCCCATTTTTG
B26	CGGATGGCACGAGAATGACCATAATCGTTTACCAGACGAC
B27	TAATTGCTTGGAAGTTTCATTCCAAATCGGTTGTA
B28	GATAAAAACCAAAATATTAAACAGTTCAGAAATTAGAGCT
B29	ACTAAAGTACGGTGTCGAATATAA
B30	GCTGTAGATCCCCCTCAAATGCTGCGAGAGGCTTTTGCA
Biotin-B30	Biotin-TTTTTGCTGTAGATCCCCCTCAAATGCTGCGAGAGGCTTTTGCA
B31	AAAGAAGTTTTGCCAGCATAAATATTCATTGACTCAACATGTT
B32	AATACTGCGGAATCGTAGGGGGTAATAGTAAAATGTTTAGACT
B32-overhang1	AATACTGCGGAATCGTAGGGGGTAATAGTAAAATGTTTAGACTGTCAGTCAGTC AGTCAGTCA
B33	AGGGATAGCTCAGAGCCACCACCCCATGTCAA
B34	CAACAGTTTATGGGATTTTGCTAATCAAAAGG
B35	GCCGCTTTGCTGAGGCTTGCAGGGGAAAAGGT
B36	GCGCAGACTCCATGTTACTTAGCCCGTTTTAA
B37	ACAGGTAGAAAGATTCATCAGTTGAGATTTAG
B38	CCTCAGAACCGCCACCCAAGCCCAATAGGAACGTAAATGA
B39	ATTTTCTGTCAGCGGAGTGAGAATACCGATAT
B40	ATTCGGTCTGCGGGATCGTCACCCGAAATCCG
B41	CGACCTGCGGTCAATCATAAGGGAACGGAACAACATTATT
B42	AGACGTTACCATGTACCGTAACACCCCTCAGAACCGCCAC

B43	CACGCATAAGAAAGGAACAACTAAGTCTTTCC
B44	ATTGTGTCTCAGCAGCGAAAGACACCATCGCC
B45	TTAATAAAACGAACTAACCGAACTGACCAACTCCTGATAA
B46	AGGTTTAGTACCGCCATGAGTTTCGTCACCAGGATCTAAA
B47	GTTTTGTCAGGAATTGCGAATAATCCGACAAT
B48	GACAACAAGCATCGGAACGAGGGTGAGATTTG
B48-overhangs2	GACAACAAGCATCGGAACGAGGGTGAGATTTGTTTTTTTTCTCTGGTTAACGTG TCTGGGC
B49	ATCATCGTTGAAAGAGGACAGATGGAAGAAAAATCTACG
Biotin-B49	Biotin-TTTTTATCATCGTTGAAAGAGGACAGATGGAAGAAAAATCTACG
B50	AGCGTAACTACAAACTACAACGCCTATCACCGTACTCAGG
B51	TAGTTGCGAATTTTTTCACGTTGATCATAGTT
B52	GTACAACGAGCAACGGCTACAGAGGATACCGA
B53	ACCAGTCAGGACGTTGGAACGGTGTACAGACCGAAACAAA
B54	ACAGACAGCCCAAATCTCCAAAAAAAAATTTCTTA
B55	AACAGCTTGCTTTGAGGACTAAAGCGATTATA
B56	CCAAGCGCAGGCGCATAGGCTGGCAGAACTGGCTCATTAT
B57	CGAGGTGAGGCTCCAAAAGGAGCC
B58	ACCCCCAGACTTTTTCATGAGGAACTTGCTTT
B59	ACCTTATGCGATTTTATGACCTTCATCAAGAGCATCTTTG
B60	CGGTTTATCAGGTTTCCATTAAACGGGAATACACT
B61	AAAACACTTAATCTTGACAAGAACTTAATCATTGTGAATT
Biotin-B61	Biotin-TTTTAAAACACTTAATCTTGACAAGAACTTAATCATTGTGAATT
B62	GGCAAAAGTAAAATACGTAATGCC
B63	TGGTTTAATTTCAACTCGGATATTCATTACCCACGAAAGA
B64	ACCAACCTAAAAAATCAACGTAACAAATAAATTGGGCTTGAGA
B65	CCTGACGAGAAACACCAGAACGAGTAGGCTGCTCATTCAGTGA
C01	TCGGGAGATATACAGTAACAGTACAAATAATT
C02	CCTGATTAAAGGAGCGGAATTATCTCGGCCTC
C03	GCAAATCACCTCAATCAATATCTGCAGGTCGA
C04	CGACCAGTACATTGGCAGATTCACCTGATTGC
Biotin-C04	Biotin-TTTTCGACCAGTACATTGGCAGATTCACCTGATTGC
C05	TGGCAATTTTTAACGTCAGATGAAAACAATAACGGATTCG
C05-Poly(T)$_{12}$	TGGCAATTTTTAACGTCAGATGAAAACAATAACGGATTCG(T)$_{12}$
C06	AAGGAATTACAAAGAAACCACCAGTCAGATGA
C07	GGACATTCACCTCAAATATCAAACACAGTTGA
C08	TTGACGAGCACGTATACTGAAATGGATTATTTAATAAAAG
C09	CCTGATTGCTTTGAATTGCGTAGATTTTCAGGCATCAATA
C10	TAATCCTGATTATCATTTTGCGGAGAGGAAGG
C11	TTATCTAAAGCATCACCTTGCTGATGGCCAAC
C12	AGAGATAGTTTGACGCTCAATCGTACGTGCTTTCCTCGTT
C13	GATTATACACAGAAATAAAGAAATACCAAGTTACAAAATC
C14	TAGGAGCATAAAAGTTTGAGTAACATTGTTTG
C15	TGACCTGACAAATGAAAAATCTAAAATATCTT

C16	AGAATCAGAGCGGGAGATGGAAATACCTACATAACCCTTC
C17	GCGCAGAGGCGAATTAATTATTTGCACGTAAATTCTGAAT
C18	AATGGAAGCGAACGTTATTAATTTCTAACAAC
C19	TAATAGATCGCTGAGAGCCAGCAGAAGCGTAA
C19-overhangs2	TAATAGATCGCTGAGAGCCAGCAGAAGCGTAATTTTTTTTCTCTGGTTAACGTG TCTGGGC
C20	GAATACGTAACAGGAAAAACGCTCCTAAACAGGAGGCCGA
Biotin-C20	Biotin-TTTTGAATACGTAACAGGAAAAACGCTCCTAAACAGGAGGCCGA
C21	TCAATAGATATTAAATCCTTTGCCGGTTAGAACCT
C22	CAATATTTGCCTGCAACAGTGCCATAGAGCCG
C23	TTAAAGGGATTTTAGATACCGCCAGCCATTGCGGCACAGA
C24	ACAATTCGACAACTCGTAATACAT
C25	TTGAGGATGGTCAGTATTAACACCTTGAATGG
C26	CTATTAGTATATCCAGAACAATATCAGGAACGGTACGCCA
C27	CGCGAACTAAAACAGAGGTGAGGCTTAGAAGTATT
C28	GAATCCTGAGAAGTGTATCGGCCTTGCTGGTACTTTAATG
C29	ACCACCAGCAGAAGATGATAGCCC
C30	AAAACATTAGAAGAACTCAAACTTTTTATAATCAGTGAG
Biotin-C30	Biotin-TTTTTAAAACATTAGAAGAACTCAAACTTTTTATAATCAGTGAG
C31	GCCACCGAGTAAAAGAACATCACTTGCCTGAGCGCCATTAAAA
C32	TCTTTGATTAGTAATAGTCTGTCCATCACGCAAATTAACCGTT
C32-overhang1	TCTTTGATTAGTAATAGTCTGTCCATCACGCAAATTAACCGTTGTCAGTCAGTCA GTCAGTCA
C33	CGCGTCTGATAGGAACGCCATCAACTTTTACA
C34	AGGAAGATGGGGACGACGACAGTAATCATATT
C35	CTCTAGAGCAAGCTTGCATGCCTGGTCAGTTG
C36	CCTTCACCGTGAGACGGGCAACAGCAGTCACA
C37	CGAGAAAGGAAGGGAAGCGTACTATGGTTGCT
C38	GCTCATTTTTTAACCAGCCTTCCTGTAGCCAGGCATCTGC
C39	CAGTTTGACGCACTCCAGCCAGCTAAACGACG
C40	GCCAGTGCGATCCCCGGGTACCGAGTTTTTCT
C41	TTTCACCAGCCTGGCCCTGAGAGAAAGCCGGCGAACGTGG
C42	GTAACCGTCTTTCATCAACATTAAAATTTTTGTTAAATCA
C43	ACGTTGTATTCCGGCACCGCTTCTGGCGCATC
C44	CCAGGGTGGCTCGAATTCGTAATCCAGTCACG
C45	TAGAGCTTGACGGGGAGTTGCAGCAAGCGGTCATTGGGCG
C46	GTTAAAATTCGCATTAATGTGAGCGAGTAACACACGTTGG
C47	TGTAGATGGGTGCCGGAAACCAGGAACGCCAG
C48	GGTTTTCCATGGTCATAGCTGTTTGAGAGGCG
C48-overhangs2	GGTTTTCCATGGTCATAGCTGTTTGAGAGGCGTTTTTTTTCTCTGGTTAACGTGT CTGGGC
C49	GTTTGCGTCACGCTGGTTTGCCCCAAGGGAGCCCCCGATT
Biotin-C49	Biotin-TTTTGTTTGCGTCACGCTGGTTTGCCCCAAGGGAGCCCCCGATT
C50	GGATAGGTACCCGTCGGATTCTCCTAAACGTTAATATTTT
C51	AGTTGGGTCAAAGCGCCATTCGCCCCGTAATG

C52	CGCGCGGGCCTGTGTGAAATTGTTGGCGATTA
C53	CTAAATCGGAACCCTAAGCAGGCGAAAATCCTTCGGCCAA
C54	CGGCGGATTGAATTCAGGCTGCGCAACGGGGGATG
C55	TGCTGCAAATCCGCTCACAATTCCCAGCTGCA
C56	TTAATGAAGTTTGATGGTGGTTCCGAGGTGCCGTAAAGCA
C57	TGGCGAAATGTTGGGAAGGGCGAT
C58	TGTCGTGCACACAACATACGAGCCACGCCAGC
C59	CAAGTTTTTTGGGGTCGAAATCGGCAAAATCCGGGAAACC
C60	TCTTCGCTATTGGAAGCATAAAGTGTATGCCCGCT
C61	CCAGTCCTTATAAATCAAAAGAGAACCATCACCCAAAT
Biotin-C61	Biotin-TTTTTTCCAGTCCTTATAAATCAAAAGAGAACCATCACCCAAAT
C62	GCGCTCACAAGCCTGGGGTGCCTA
C63	CGATGGCCCACTACGTATAGCCCGAGATAGGGATTGCGTT
C64	AACTCACATTATTGAGTGTTGTTCCAGAAACCGTCTATCAGGG
C65	ACGTGGACTCCAACGTCAAAGGGCGAATTTGGAACAAGAGTCC
Link-A1C	TTAATTAATTTTTTACCATATCAAA
Link-A2C	TTAATTTCATCTTAGACTTTACAA
Link-A3C	CTGTCCAGACGTATACCGAACGA
Link-A4C	TCAAGATTAGTGTAGCAATACT
Link-B1A	TGTAGCATTCCTTTTATAAACAGTT
Link-B2A	TTTAATTGTATTTCCACCAGAGCC
Link-B3A	ACTACGAAGGCTTAGCACCATTA
Link-B4A	ATAAGGCTTGCAACAAAGTTAC
Link-C1B	GTGGGAACAAATTTCTATTTTTGAG
Link-C2B	CGGTGCGGGCCTTCCAAAAACATT
Link-C3B	ATGAGTGAGCTTTTAAATATGCA
Link-C4B	ACTATTAAAGAGGATAGCGTCC
Loop	GCGCTTAATGCGCCGCTACAGGGC

Table S2. The sequences of oligonucleotides for the cross DNA origami used in this work

Name	The sequences (from 5' to 3')
1	CGTGAACCATCACCCAAATCAAGTGCCGTAAA
2	GAAAAACCGTCTATCAGGGCGATGAGCCCCCG
3	ATTAAAGAACGTGGACTCCAACGTGGCGAACG
4	GTTCCAGTTTGGAACAAGCGAAAGGA
5	GTCGAGGTTTTTTGGGTAGTAAAAGGGGGTAA
6	GCACTAAATAACGTGCTTTCCTCGAGCTAAAC
7	ATTTAGAGGCGCGTACTATGGTTGGACAGGAA
8	TGGCGAGACACACCCGCCGCGCTTGTTTTTAT
9	GCGGGCGCTAGGGTTTTAGTCGGGAAA
10	AGCACGTATCGGAACCCTAAAGGGGCCCACTA
11	GCTACAGGCTTGACGGGGAAAGCCCAAAGGGC
Poly(T)$_{12}$-11	(T)$_{12}$TTTTGCTACAGGCTTGACGGGGAAAGCCCAAAGGGC

12	GTAACCACAAGGAAGGGAAGAAAGAGTCCACT
13	AGCGGTCAGAGTCTGTCCATCACG
14	AGAGCGGGTTAGAATCTACCAGTCGCTCATTA
15	AGGAGGCCATTTTGACGCTCAATCTTACATTG
16	CGGTACGCGCAACAGGAAAAACGCGTAATAAA
17	AATCAGTGTAATATCCAGAACAATAGAACCCT
18	GTAGCAATACTTCTTTTTATTACGCCAGTTGCGTTGCGCTCACT
19	ATACCTACGATTAAAGGGATTTTACTTTGACG
20	CAGCCATTCAGAATCCTGAGAAGTAATGCGCC
21	CTTGCTGGAGGCCACCGAGTAAAACGCTGCGC
22	AGTAGAAGAACTCAAAGTGGCACAGACAATAT
23	TAACATCACTTGCCTGCAAATTAACCGTT
23-Poly(T)$_{12}$	TAACATCACTTGCCTGCAAATTAACCGTT(T)$_{12}$
24	TTGATTAGTAAGTCTTTAATGCTTTGATCGGTGCGGGCCTCAACTGTTGGGAAG GGC
25	TGGATTATGTCTGAAACGGAACGATACTTAGC
26	GCAGATTCTATCAAACCCTCAATCGCAAATCA
27	AGGGACATAAATCTAAAGCATCACTTATCTAA
28	TCTGACCTCAGTGCCACGCTGAGATAATAGAT
29	GCGAACCGAA
30	ACCTCAAAACCAGTCACACGACCATCATGGAA
31	CAAATGAATCTGGCCAACAGAGATATTACCGC
32	GCCTGCAAGAAAGCGTAAGAATACCTATCGGC
33	GGTGAGGCGGTCAGTATTGAGGATTTAGAAGT
34	CAGAAGATAAAACAGATTTTGAATGGCTATTA
35	ATCGCCATTAAAAATACTGATAGCCCTAAAACTTTTGTTAAATCAGCTAAAATT CGCATTAAAT
36	ACAGTTGATGGCAATTCATCAATAGATTATAC
37	AATATCTTGAATTATCATCATATTCCTACCAT
38	TAGAGCCGTTTGCGGAACAAAGAAAGAAATAA
39	TCAGATGAAAGGAATTGAGGAAGGCTTGCTGA
40	AAGGAGCGTAGGAGCACTAACAACGCCAGCAG
41	ATTATCATTCAATAGATAATACATTTAACACC
42	ATTAATTTTAAAAGTTTAACGTCA
43	TTGCCCGAACGTTATTAGACTTTACAAAC
Poly(T)$_{12}$-43	(T)$_{12}$TTTTTTGCCCGAACGTTATTAGACTTTACAAAC
44	TTCTGAATACCTGAGCAAAAGAAGCAAGAAAA
45	ATCAAAATAATCGCGCAGAGGCGATCATTTGA
46	AGAAATTGTTCGCCTGATTGCTTTACATAAAT
47	GATGAATATACAGTTTTTTAAATAAGGCGT
48	TTTCAATTAATGGAAGGGTTAGAACCTGATTA
49	AGTTACAATATTTGCACGTAAAACACCACCAG
50	ATAACGGACGTAGATTTTCAGGTTTGAGTAAC
51	TACATCGGGCTTCTGTAAATCGTCGCTATT

52	CAAAATTAATTACATTTAACAATTATTATTCA
53	ATTACCTTTTTTAATGGAAACAGTGAATACCA
54	CAATATATGTGAGTGAATAACCTTGAGAAACA
55	TCTGAGAGACTACCTTTTTAACCTTATATAAC
56	AGAGTCAATAGTGAATTTATCAAACAATCGCA
57	ACATAGCGATAGCTTAGATTAAGATTCAAATA
58	TTAATTTTCCCTTAGAATACCTAAAT
59	GGTTGGGTCCGGCTTATGAGAGAGCCTGGCCC
60	TATATGTATAACAACGCCAACATGATTTTCGA
61	AGACAAAGAACAGTAGGGCTTAATACCGACAA
62	TATTTTAGTACAAATTCTTACCAGGACGACAA
63	TTAATGGTTAGAAAAAGCCTGTTTGAACGCGC
64	ACCGTGTGATTTTTTAACAGTACCTTT
65	GCCATATTAATGCTGATGCAAATCATCATAGG
66	CAACGCTCAACGCGAGAAAACTTTCGCTGAGA
Biotin-66	Biotin-TTTTCAACGCTCAACGCGAGAAAACTTTCGCTGAGA
67	ATGCGTTATTAATTTCATCTTCTGCCTTGAAA
68	ATAATTACTTGAAATACCG
Biotin-68	Biotin-TTTTATAATTACTTGAAATACCG
69	TAAATAAGAATAAACACTGAACAAGAATTTTCGTATTAAATCCT
70	GCAGAGGCTAATTTAGAGCTCGAAGGGTACCG
71	GCCAGTAAGCAAATCAGATATAGACTAAGAAC
72	AAGGTAAAATCGTAGGAATCATTAGACTTGCG
73	TAAACAACTCATCGAGAACAAGCAGATTAGTT
74	CTGTTTATTCCAAGAACGGGTATTTTATCCTG
75	AAATACCAATCAATAATCGGC
76	AATAGCAATAAGAGAATATAAAGTTGAGAATC
77	TTATTTTCGTAATTCTGTCCAGACTATAAAGC
78	TACCGCACATGTTCAGCTAATGCAAGTATCAT
79	CTTATCATCAACAATAGATAAGTCCCGGAATC
80	TACGAGCATGTAGAAAATATCCCATCCTAATTTTTCGAACCACCAGAATTCGACAAC
81	CCGGTATTAGGCTTATTGGTGTAGGGTCACGT
82	GCGAGGCGAAGTCAGAGGGTAATTGAGATAAC
83	GGAGGTTTTAGACGGGAGAATTAATAATAAGA
84	GCTATTTTAGAGAGAATAACATAAGCTATCTT
85	AATCTTACCAACGCTAACGAGCGTTGTCTTTC
86	CTTTCCAGAGCCTAATTATCCCAATCCAAATA
87	CCTGAACATTTTAGCGAACCTCCCCCGCGCCC
88	AAGCGCATTGAAGCCTTAAATCAAAGCCGTTT
89	GCCTTTACGCACCCAGCTACAATTAAACCAAG
90	TTTTTTGTTTAACGTCAAAAATGAAAGCAGAT
91	CCACAAGAATGGTTTACCAGCGCCCATTCAAC
92	GCAAGAAAGTTTATTTTGTCACAATTGACGGA

93	ACCGAAGCTATAAAAGAAACGCAACACCGTCA
94	AGCCGAACACGTAGAAAATACATAGAGCCAGC
95	AACCGAGGAAACGCAATTAAG
96	AAATTCATATTGAGTTAAGCCCAACTGAACAC
97	CGGAATAACAATGAAATAGCAATAAAACAGGG
Biotin-97	Biotin-TTTTCGGAATAACAATGAAATAGCAATAAAACAGGG
98	TGGCAACACCTTTTTAAGAAAAGTAAATAGCA
99	GTTAGCAAAAAGTTACCAGAAGGAAGAAACGA
Biotin-99	Biotin-TTTTGTTAGCAAAAAGTTACCAGAAGGAAGAAACGA
100	CGATTGAGCATCTTTTCATAATCAAGCCACCA
101	AATTATTCTTTTCGGTCATAGCCCCCACCCTC
102	CCGACTTGTAGCGTCAGACTGTAGACCCTCAG
103	AAAATCACGTAATCAGTAGCGACAAGCCGCCGCCAGCATTGACAGG
104	GCGTTTGCGGAGGGAAGGTAAATATCAATAGA
105	CATCGGCAATTAAAGGTGAATTATAGACACCA
106	TTTGCCTTAGCCATTTGGGAATTACATAAAGG
107	ATAGCAGCACCCAGTAGCACCATTACCCGCAGTAT
108	ACGTCACCAATTTTTTTCCCTCAGAGCCAC
109	CCGGAACCGCCTCCCTCAGAGCCGCCTTATTA
110	AGAACCGCCACCCTCAGAGCCACCCGCGTTTT
111	AGCCGCCACCAGAACCACCACCAGGAATCAAG
112	CGTTCCAGTAAGCGTCGGATTAGCCCAGGCGG
113	GCCAGAATGGAAAGCGCAGTCTCTATAAGTAT
114	TGATATTCACAAACAAATAAATCCACTCAGGA
115	GTTGAGGCAGGTCAGACGCCGCCACC
116	GCTCAGTAGGGGTTTTACAAACATATGATGAA
117	ATAAGTGCAGCATTCCACAGACAGAACGATCT
118	AGCCCGGAGTTTCGTCACCAGTACTTAGTAAA
119	GGTTTAGTGCCCAATAGGAACCCATAAACAAC
120	CTCAGAACCGCCATTTTGAAACCATCG
121	ACGCCTGTCGTCGAGAGGGTTGATGAATTTAC
122	AACACTGAATAGGTGTATCACCGTTCATTAAA
Biotin-122	Biotin-TTTTAACACTGAATAGGTGTATCACCGTTCATTAAA
123	GATAGCAAACCGCCACCCTCAGAAATTGGCCT
124	CACCCTCAAATAGAAAGGAACAAC
125	GTTAGCGTCCCTCATAATTGTTTGTAATCCTG
126	AAAGTTTTCAACCATCGCCCACGCGTCGCTGA
127	TGAATTTTCTTGATACCGATAGTTTTTTGCGG
128	TTTCAACACAGCTTGCTTTCGAGGACAGCATC
129	AATAATAATTTTTTTTTACTCCTTATTAATTAGCAAGGCCGGAA
130	AATGACAAGTCGTCTTTCCAGACGAAACTACA
131	TTAAACAGCTGTATGGGATTTTGCTGTACCGT
132	CGGTTTATGTTTCAGCGGAGTGAGTTTTCAGG
133	TCCAAAAGGAGCCTTTGAGGCTTTGAGGACTA

134	TCTCCAAAAAAAAGGCTAAAGGAATTGCG
134-Biotin	TCTCCAAAAAAAAGGCTAAAGGAATTGCGTTTT-Biotin
135	CACGTTGAAAAGAAGTTTCCATTTTAAAAGAACTGGCATGATAATAACGGAATACCC
136	TATATTCGATAACCGAGTCAGTTGAATATCTG
137	GGCTTGCAGCGACCTGCTCCATGTGGCGCAGA
138	GATCGTCAGTATCATCGCCTGATATGACCAAC
139	GGAACGAGACCAAGCGCGAAACAAGTACAGAC
140	TAAACCAACC
141	CGAAATCCGGGAGTTAAAGGCCGCGCGCCGAC
142	GGAGATTTCCCTCAGCAGCGAAAGTGAATTTC
143	GCGATTATGGTAGCAACGGCTACAAATTGTAT
144	TAAAACACTCATCTTTCATCAAGAGTAATCTT
145	AGGCAAAAGAATACACAAGACTTTTTCATGAG
146	GCCACTACGAAGGCACGGGTAAAATACGTAATAAACAGCCATATTATTTTGCCAGTTACAAAAT
147	CGGTCAATGCGATTTTAAGAACTGAGGACGTT
148	TTTGAAAGATTTCAACTTTAATCAAACGAACT
149	CAGGCGCAAACGAGTAGTAAATTGGAAAGATT
150	TACCTTATCATAAGGGAACCGAACAATTGTGT
151	ATGGTTTAAGGACAGATGAACGGTAGTACAAC
Biotin-151	Biotin-TTTTATGGTTTAAGGACAGATGAACGGTAGTACAAC
152	AACACCAGTAGGCTGGCTGACCTTGACCCCCA
153	TGAATAAGGCTTGCCCACATTCAA
154	GCTGCTCATTCAGGACAAGAACCGGATAT
154-Biotin	GCTGCTCATTCAGGACAAGAACCGGATATTTTT-Biotin
155	GGGAAGAAAAGAAGTTTTGCCAGATGTTTAGA
156	AACGGAACATAAAAACCAAAATAGATCGTCAT
157	CATCAGTTACACTATCATAACCCTATGCTTTA
158	CTAATGCAGATACTTTTTTAGTTTCATTCC
159	TTTTGCAAAAATCTACGTTAATAATTGTGAAT
160	AGACGACGAACATTATTACAGGTAGGCTTGAG
161	TAAGAGCAGAGATTTAGGAATACCTGACGAGA
162	GAATTACGCATAAATCAAAAATCAGGTCTT
163	CTGGATAGCGTCCAATACTGCGGACGAGAGGC
164	AAATATTCATTGAATCCCCCTCAACGTTTACC
165	AACAGTTCAGAAAACGAGAATGACAGGCATAG
166	GCTTCAAAGCGAACCAGACCGGAACAGGATTA
167	CGAAAGACTTCAAATATCGCGTTTTGATAAGA
168	GCGGATTGCATCAAAAAGATTAAGGCTTAATT
169	CCCTGACTATTATAGTCACATGTTTT
170	CAACAGGTGCAAACTCGGAACCAGAAATCACC
171	GAGAGTACATTCTACTAATAGTAGTAAATCAT
172	GGTCATTTCATTTGGGGCGCGAGCAATTAAGC

173	GCTGAATACAAATGGTCAATAACCAAATCGGT
174	AAATATGCGTAGATTTAGTTTGACTAATACTT
175	GGTGTCTGGATTTTATAACGCCAAAAG
176	TGGCATCACTTTAATTGCTCCTTTTAATTCGA
177	TATATTTTTTGCGGATGGCTTAGAAGGAAGCC
Biotin-177	Biotin-TTTTTATATTTTTTGCGGATGGCTTAGAAGGAAGCC
178	ACATTTCGTAATGCTGTAGCTCAAGAAGCAAA
179	GCGAACGAAACTAAAGTAC
Biotin-179	Biotin-TTTTGCGAACGAAACTAAAGTAC
180	ATATAACAGTTGATTCCGCAAGGATAATTTATCAACGTAACAAA
181	ACATCCAATAGCATTAAAGGGCGAAAAGACAA
182	ACAGGCAATCTACAAAGGCTATCATCTGGAGC
183	AATAAAGCTAATGCCGGAGAGGGTATCGTAAA
184	TGTACCAAAATATGATATTCAACCCCGGTTGA
185	TTGCGGGATGAGAAAGGCCGGAGAGAAGATTG
186	AAATTCTGAGTAATGTGTAGG
187	TTGAGAGAGGCAAAGAATTAGCAATGAAAAGG
188	TGATAAATCTCAGAGCATAAAGCTTGTTTAGC
189	TCACCATCAAACATTATGACCCTGCATTAGAT
190	CAAAAGGGGAAGCCTTTATTTCAACCAATTCT
191	ATTTTAAATGCAATGCTTTAGAACCCTCATATTTTTAAAACGAAAGTCATTACCC AA
192	CCTGAGAGGGTCATTGATATCAGAGAGCGCTA
193	AAACAAGATGACCGTAATGGGATAATGGGCGC
194	ACTAGCATCGTCGGATTCTCCGTGTGAGGGGA
195	TAATCAGATCATCAACATTAAATGGATCGCAC
196	TATAAGCAAATATTTAAATTGTAATAAAGATT
197	ACGTTAATATTTTGTTCATTTTTTAACCAATA
198	CGGCGGATGAATCGATGAACGGTAAGCTATTT
199	TAACAACCGTCAATCATATGTACCGTTCTAGC
200	GCCAGCTTAAAGCCCCAAAAACAGCAGTCAAA
201	ATCAAAAATAATTCGCGTCTGGCCTCTGGTGC
202	ATCGTAACACTCTAGAGGATCCCCTTCGTAAT
203	CGACGACAGGCCAGTGCCAAGCTTGAAATTGT
204	TCCAGCCAGGGTTTTCCCAGTCACATACGAGC
205	CGGAAACCGTGCTGCAAGGCGATTGGGTGCCT
206	GCCATTCAGGCTGCGCTTCGC
207	GCAGGTCGCGTGCATCTGCCAGTTGGAACAAA
208	AAAACGACGTATCGGCCTCAGGAATGAGCGAG
Biotin-208	Biotin-TTTTAAAACGACGTATCGGCCTCAGGAATGAGCGAG
209	TAACGCCAGCTTTCCGGCACCGCTTTCCTGTA
210	AGGGGGATAGGCAAAGCGCCATTCGGAACGCC
Biotin-210	Biotin-TTTTAGGGGGATAGGCAAAGCGCCATTCGGAACGCC
211	CATGGTCATGATTGCCCTTCACCGTTGCAGCA

212	TATCCGCTTTTTTCTTTTCACCAGGCAGGCGA
213	CGGAAGCAAGAGGCGGTTTGCGTAAAATCGGC
214	AATGAGTGAGCTGCATTAATGAATTAGCCCGAGATAGGGTTGAGTG
215	GCAACAGCTAGCTGTTTCCTGTGTGCATGCCT
216	CAGGGTGGCACAATTCCACACAACGACGTTGT
217	GCGCGGGGTAAAGTGTAAAGCCTGAAGTTGGG
218	CCTGTCGTGCCAGCTAACTCACATTAACTGGCGAA
219	GCCCGCTTTCCTTTTTTCGCTGGCAAGTGT
220	AGCGGTCCACGCTGGTTTGCCCCATGAGACGG
221	AAATCCTGTTTGATGGTGGTTCCGTTGGGCGC
222	AAAATCCCTTATAAATCAAAAGAACGGCCAAC
